# Succinate Dehydrogenase loss causes cascading metabolic effects that impair pyrimidine biosynthesis

**DOI:** 10.1101/2025.02.18.638948

**Published:** 2025-02-19

**Authors:** Madeleine L. Hart, Kristian Davidsen, Serwah Danquah, Eric Zheng, David Sokolov, Lucas B. Sullivan

**Affiliations:** 1 Human Biology Division, Fred Hutchinson Cancer Center, Seattle, WA, 98109, USA.

**Keywords:** aspartate, biosensor, metabolism, SDH, proliferation, metabolomics, pyrimidines, cancer

## Abstract

Impaired availability of the amino acid aspartate can be a metabolic constraint of cell proliferation in diverse biological contexts. However, the kinetics of aspartate depletion, and its ramifications on downstream metabolism and cell proliferation, remain poorly understood. Here, we deploy the aspartate biosensor jAspSnFR3 with live cell imaging to resolve temporal relationships between aspartate and cell proliferation from genetic, pharmacological, and nutritional manipulations. In cells with impaired aspartate acquisition from mitochondrial complex I inhibition or constrained uptake in aspartate auxotrophs, we find that the proliferation defects lag changes in aspartate levels and only manifest once aspartate levels fall below a critical threshold, supporting the functional link between aspartate levels and cell proliferation in these contexts. In another context of aspartate synthesis inhibition, impairing succinate dehydrogenase (SDH), we find a more complex metabolic interaction, with initial aspartate depletion followed by a rebound of aspartate levels over time. We find that this aspartate rebound effect results from SDH inhibition disproportionately impairing pyrimidine synthesis by inhibiting aspartate transcarbamoylase (ATCase) through the dual effect of diminishing aspartate substrate availability while accumulating succinate, which functions as a competitive inhibitor of aspartate utilization. Finally, we uncover that the nucleotide imbalance from SDH inhibition causes replication stress and introduces a vulnerability to ATR kinase inhibition. Altogether, these findings identify a mechanistic role for succinate in modulating nucleotide synthesis and demonstrate how cascading metabolic interactions can unfold to impact cell function.

## Introduction:

Cell metabolism is amongst the most dynamic cellular processes, with biochemical reactions operating at the sub-second timescale and the turnover of metabolic pathways occurring within seconds to hours. Metabolic disruptions can therefore have complex effects over time, as metabolic changes shift the abundance of linked metabolites, causing reverberations across the metabolic network. While conventional metabolite quantification methods like mass spectrometry-based metabolomics enable precise and accurate measurements of many metabolites within a sample, they also necessitate destructive metabolite extraction protocols, limiting throughput. As a result, limited time points are typically used in metabolomics experiments and the intervening effects on metabolite levels are typically extrapolated between them, which may or may not be appropriate. One approach to overcome this challenge is to measure metabolite levels with non-toxic reporters, such as genetically encoded biosensors. At the expense of measuring fewer metabolic variables, these tools can be coupled with live cell imaging techniques to monitor changes in metabolite levels over frequent time points, near simultaneously across dozens of conditions^1^. The use of live cell imaging also provides an opportunity to frequently measure cell number, which can then be used to calculate cell proliferation rates, another variable that is often extrapolated from limited time points for similar reasons. While the availability of specific metabolite biosensors has historically limited the utility of this approach, recent efforts have substantially increased the repertoire of metabolite biosensors, including one that we recently described to measure intracellular aspartate (jAspSnFR3)^2–7^. We thus hypothesized that deploying biosensors and live cell imaging will provide an important opportunity to coordinate metabolic changes with their impact on cell proliferation.

Intracellular aspartate abundance is a particularly important metabolic variable as it is both readout of cellular metabolic state and a critical determinant of cell function. In humans and mice, aspartate has amongst the lowest concentrations of all the amino acids in circulation and most cells do not express a transporter for aspartate, making them dependent on *de novo* synthesis. Aspartate is produced by transamination of the tricarboxylic acid (TCA) cycle intermediate oxaloacetate (OAA) by either the enzyme GOT1 (cytosolic) or GOT2 (mitochondrial). OAA can be derived from several nutrient sources that each use diverse metabolic routes across multiple cellular compartments - each with their own regulation and metabolic costs. Since the production of aspartate integrates so many metabolic variables, aspartate abundance is susceptible to changes from diverse metabolic perturbations. For example, impairments to complexes I, III, IV, and V of the mitochondrial electron transport chain (ETC) can decrease cellular NAD+/NADH levels and thereby slow the production of TCA cycle derived aspartate^8–11^. In addition, the inhibition of succinate dehydrogenase (SDH, also known as ETC complex II) directly impairs TCA cycle derived aspartate production, without the requirement for decreased NAD+/NADH^12–15^. Genetic knockout of GOT1, GOT2, or both can also diminish aspartate levels in various metabolic contexts^2,9,16–19^. Aspartate levels are also impacted by more complex metabolic perturbations, such as hypoxia and glutamine limitation^16,20–23^. Notably, all of these perturbations have been found to also impair cell proliferation in a manner that is ameliorated by restoring aspartate levels^8–12,16–19,21,23,24^. Collectively, we refer to the phenomenon where aspartate metabolism is impacted by a perturbation, causing an impairment in cell function that can be ameliorated by aspartate restoration – as “aspartate limitation.” However, it remains unclear how aspartate levels change over time in these contexts and how aspartate depletion impacts downstream metabolic fates and cell proliferation. Since aspartate limitation has been observed in diverse physiological contexts, dynamic understanding of how aspartate levels change and mediate their metabolic and functional consequences is critical to better appreciate this important metabolic phenomenon.

In this manuscript, we employ our recently described intracellular aspartate biosensor jAspSnFR3 and live cell imaging to explore the relationship between aspartate levels and proliferation rate across multiple models of aspartate limitation. Our findings demonstrate that this bioassay can competently and non-destructively measure aspartate levels and cell proliferation rates at substantially shorter intervals compared to conventional methods across multiple treatment conditions. These measurements reveal that aspartate limiting treatments cause dynamic effects on aspartate levels and proliferation rates, with consequences on cell proliferation occurring once aspartate levels are sufficiently depleted. Investigating the effects of SDH inhibition, we find variable aspartate levels over time, a phenomenon that revealed a complex interaction where aspartate depletion collaborates with succinate accumulation to impair *de novo* pyrimidine synthesis. Finally, we find that the effects of SDH inhibition on nucleotide biosynthesis cause replication stress, resulting in a vulnerability to ATR inhibition.

These results therefore provide novel insights into the complex interactions between aspartate levels and cellular proliferation that would otherwise be difficult to discover using standard techniques.

## Results:

### Limiting aspartate acquisition causes delayed effects on cell proliferation that manifest upon aspartate depletion

To enable simultaneous measurements of aspartate levels and cell proliferation by live cell imaging we generated single cell clones of 143B and H1299 cells expressing a cytosol localized aspartate sensor (jAspSnFR3) and a nuclear localized variant of the red fluorescent protein mRuby2 (NucRFP). In this system, aspartate abundance is reported by jAspSnFR3 through fluorescence intensity in the green fluorescence channel and normalized to RFP intensity (GFP/RFP) to account for changes in fluorescent protein expression and cell number^2^. An additional advantage to this system is that NucRFP expression enables facile cell counting during microscopy by leveraging the distinct boundaries of fluorescent nuclei to measure RFP object instances (nuclei counts). With these two outputs able to be measured multiple times per day using live cell imaging, we can measure the relationship between aspartate levels and cell proliferation at unprecedented detail.

**Figure 1.**
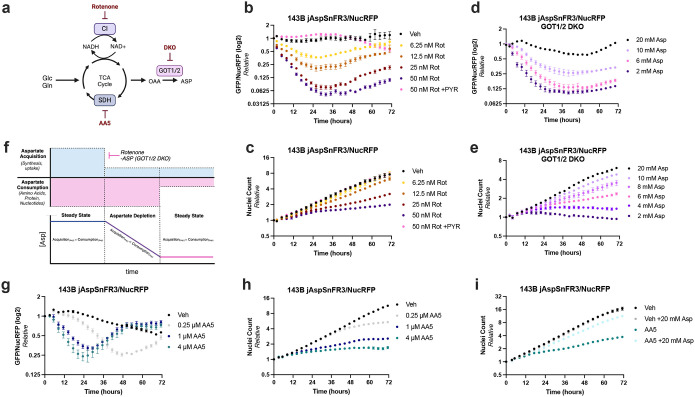
Temporal measurements of aspartate levels and cell proliferation upon treatments that constrain aspartate acquisition reveals distinct patterns. a. Model depicting three methods of inducing aspartate limitation in cells. Complex I inhibition with rotenone blocks NAD+ regeneration thereby slowing TCA cycling; GOT1/2 double knockout (DKO) blocks transamination of oxaloacetate (OAA) to generate aspartate; and atpenin A5 (AA5) inhibits succinate dehydrogenase (SDH) activity, directly blocking the oxidative TCA cycle upstream of aspartate synthesis. b. GFP/RFP ratio of 143B jAspSnFR3/NucRFP cells treated with a rotenone titration, with one condition cultured with 1 mM pyruvate (+PYR), in DMEM without pyruvate (n=4). c. NucRFP counts per well of 143B jAspSnFR3/NucRFP cells treated with a rotenone titration, with one condition cultured with 1 mM pyruvate in DMEM without pyruvate (n=4). d. GFP/RFP ratio of 143B jAspSnFR3/NucRFP GOT1/2 double knockout (DKO) cells against a titration of environmental aspartate concentrations in DMEM without pyruvate (n=4). e. NucRFP counts of 143B jAspSnFR3/NucRFP GOT1/2 double knockout (DKO) cells against a titration of environmental aspartate concentrations in DMEM without pyruvate (n=4). f. Model showing that aspartate acquisition and consumption are basally matched at steady state until acquisition is inhibited by either disrupting synthesis through complex I impairment (Rotenone) in WT cells or by double knockout (DKO) of GOT1 and GOT2 DKO) and slowing uptake by environmental aspartate withdrawal (-ASP). Following aspartate acquisition impairments, aspartate levels decay while the rate of aspartate consumption for biosynthesis (measured as cell proliferation) is maintained. When the aspartate pool is depleted to the point that it slows aspartate consumption, cells enter a new steady state at a lower aspartate level, where acquisition and consumption are slowed, but once again matched. g. GFP/RFP ratio of 143B jAspSnFR3/NucRFP cells treated with an AA5 titration in DMEM (n=4). h. NucRFP counts of 143B jAspSnFR3/NucRFP cells treated with an AA5 titration in DMEM (n=4). i. NucRFP counts of 143B jAspSnFR3/NucRFP cells treated with either Veh or 5 μM AA5 with or without 20 mM aspartate in DMEM (n=3). Data are plotted as means ± standard deviation (SD) except g and h which are shown as means ± standard error of the mean (SEM).

To validate our bioassay in a conventional setting of aspartate limitation, we first subjected jAspSnFR3/NucRFP 143B and H1299 cancer cells to a titration of the ETC complex I inhibitor rotenone. Complex I inhibition blocks NAD+ regeneration from NADH, thereby slowing NAD+ dependent reactions in the TCA cycle and impairing aspartate synthesis (Fig. 1a). Consistent with previous findings, we observed that rotenone causes a dose-dependent decrease in aspartate levels and cell proliferation, both of which can be prevented by co-treatment with pyruvate, a metabolic rescue that orthogonally restores NADH oxidation (Fig. 1b–c, Extended Data Fig. 1a–b)^2,8,10,12^. Temporally, rotenone treatment caused a rapid decay in aspartate levels which stabilized at a lower abundance after around 24 hours, dose dependently corresponding to rotenone concentration. In contrast, the impacts of rotenone treatment on cell proliferation were not immediate and were only impactful at around the same 24-hour timepoint. After this timepoint, rotenone treated cells settled into a new pseudo-steady state of lower aspartate levels and correspondingly slower cell proliferation rates (Fig. 1b–c, Extended Data Fig. 1a–b)^2^. Importantly, these findings were verified using the traditional approaches of LC-MS metabolomics for metabolite levels and cell counts for proliferation rates, confirming the accuracy of this high throughput approach (Extended Data Fig. 1c–d). While there was a small increase in both aspartate and cell proliferation in rotenone treated cells after two days, this effect may result from degradation of intracellular rotenone over time, which was observed by LCMS, and/or activation of alternative aspartate synthesis pathways (Extended Data Fig. 1e). Overall, these results underscore the importance of aspartate depletion in mediating the functional effects of complex I inhibition on cell proliferation.

We next turned to a condition that introduces aspartate limitation without a pharmacological perturbation. We generated single cell clones of cells expressing jAspSnFR3 and NucRFP in previously generated 143B and H1299 cells lacking both glutamic-oxaloacetic transaminases 1 and 2 (GOT1/2 DKO), which renders them unable to synthesize aspartate (Fig. 1a, Extended Data Fig. 1f)^2^. Due to the low permeability of exogenous aspartate, GOT1/2 DKO cells are maintained in 20–40 mM aspartate to maximally support cell proliferation, in agreement with previously measured parameters for non-specific aspartate uptake^2,9,23^. As expected, subjecting GOT1/2 DKO cells to decreasing media concentrations of aspartate led to dose dependent proliferation defects, as determined by conventional cell counting (Extended Data Fig. 1g–h). When these conditions were evaluated by live cell imaging, we observed similar kinetics of aspartate depletion to what was observed in rotenone treated cells, with a rapid decay in intracellular aspartate that stabilized after approximately 24 hours (Fig. 1d, Extended Data Fig. 1i). The effects on proliferation also mirrored what was found with rotenone treated cells, where proliferation was maintained for most conditions in the first 24 hours, after which a pseudo-steady state proliferation rate emerged that was proportional to intracellular aspartate levels (Fig. 1d–e, Extended Data 1i–j). Overall, these findings affirm the utility of this approach to coordinate aspartate levels with functional effects and highlight the evolving nature by which metabolic perturbations impact metabolism and mediate their functional effects on cells.

Concentrations of intracellular metabolites reflect the balance between acquisition and consumption. Aspartate acquisition consists of either biosynthesis, the predominant route of aspartate acquisition in most settings, or uptake. Aspartate consumption is used to support multiple components of macromolecular synthesis pathways necessary for cell proliferation, meaning the aspartate consumption rate can be considered proportional to cell proliferation rate. Our experiments thus suggest a model where cells in standard conditions have matched, high levels of aspartate acquisition and aspartate consumption, leading to a stable aspartate concentration over time. Impairing aspartate acquisition initially occurs without a corresponding decrease in aspartate consumption, leading to a progressive decrease in aspartate levels as aspartate consumption is greater than aspartate acquisition. When the reservoir of aspartate falls to a level that slows aspartate consumption, presumably by impairing its usage in macromolecular synthesis during cell proliferation, a new steady state of aspartate levels emerges where aspartate levels are again relatively stable due to matched lower levels of acquisition and consumption (Fig. 1f). To further evaluate this model, we tested the converse scenario, determining if impairing cell proliferation without affecting aspartate acquisition would increase aspartate levels. Indeed, treatment of 143B cells with the protein synthesis inhibitor cycloheximide (CHX) immediately arrested cell proliferation and correspondingly increased aspartate levels (Extended Data Fig. 1k–l).

### Aspartate levels decrease, then rebound after SDH inhibition

We next used jAspSnFR3/NucRFP 143B cells to measure aspartate levels and proliferation rate when subjected to the SDH inhibitor, Atpenin A5 (AA5), which blocks aspartate synthesis by directly impairing the oxidative TCA cycle (Fig. 1a). We and others have shown that pyruvate supplementation in SDH impaired cells is required to support alternative aspartate synthesis pathways and can modestly improve cell proliferation, so we first evaluated these effects in media containing pyruvate^12,13,15^. SDH inhibition caused aspartate levels to fall, with a rate of decay proportional to AA5 dose, as in other contexts of aspartate limitation (Fig. 1g). SDH inhibition caused aspartate levels to fall, with a rate of decay proportional to AA5 dose, in agreement with other contexts of aspartate limitation (Fig. 1g). However, upon hitting a nadir, aspartate levels then rebound and ultimately reach a new steady state at an aspartate level closer to that of control cells (Fig. 1g). The functional effects on cell proliferation initially mirrored what was seen in other models of aspartate acquisition impairments, with a delayed effect on cell proliferation that manifested as an inflection point when aspartate approached its nadir (Fig. 1h). However, unlike other models of aspartate limitation, aspartate levels and cell proliferation were not coupled afterwards, since the ensuing recovery of aspartate levels in SDH inhibited cells did not coincide with a restoration of cell proliferation. Similar effects of SDH inhibition on aspartate levels and cell proliferation were also observed in media without pyruvate (Extended Data Fig. 1m–n). Notably, the lack of a proliferation rebound upon increased aspartate levels is particularly surprising when considering that proliferation is also restored to these cells when supplemented with exogenous aspartate (Fig. 1i, Extended Data Fig. 1o)^12^.

Collectively, temporal measurements of aspartate levels and cell number upon three modes of aspartate acquisition perturbations identifies two discrete phenomena: 1) A straightforward effect where impairing aspartate acquisition leads to a decline in aspartate until it becomes limiting, at which point cell proliferation slows to match the constrained aspartate acquisition rate. 2) A curious effect of SDH inhibition where aspartate levels decline and then rebound after hitting a nadir, without a commensurate restoration on cell proliferation. Given the non-intuitive dynamics of the latter response, and the importance of aspartate availability for SDH impaired cells, we next focused understanding how SDH inhibition causes this effect.

### SDH inhibition impairs pyrimidine synthesis beyond its effects on aspartate depletion

**Figure 2.**
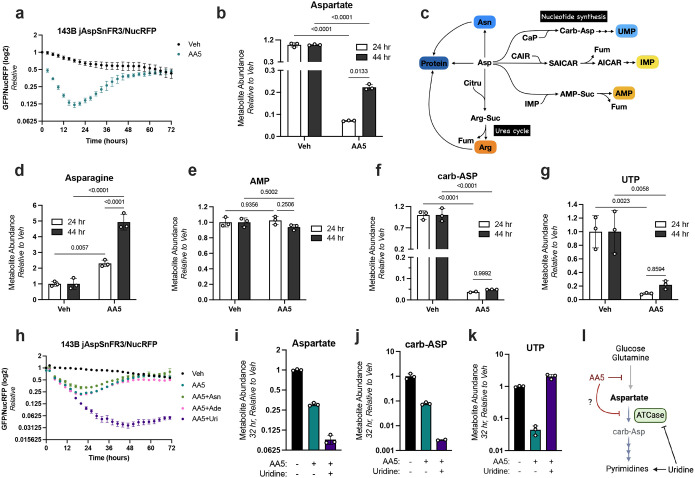
SDH inhibition causes dynamic changes in aspartate levels corresponding to emergent effects on pyrimidine synthesis. a. GFP/RFP of 143B jAspSnFR3/NucRFP cells treated with either Veh (DMSO) or 5 μM AA5 in DMEM (n=4). b. Aspartate levels measured by LC-MS of 143B cells treated with either Veh (DMSO) or 5 μM AA5 in DMEM extracted at 24 hours and 44 hours post treatment normalized to Veh (n=3). c. Model showing the metabolic fates of aspartate. Aspartate contributes directly to protein synthesis, donates carbon to the production of asparagine (Asn) and pyrimidines (UMP), and donates nitrogen during the synthesis of arginine (Arg) and purines (IMP, AMP). Abbreviations: CaP, carbamoyl-phosphate; Carb-Asp, carbamoyl-aspartate, Fum, fumarate; AMP-succ, adenylosuccinate; Citru, citrulline; Arg-Suc, argininosuccinate. d. Asparagine (Asn) levels measured by LC-MS of 143B cells treated with either Veh (DMSO) or 5 μM AA5 in DMEM extracted at 24 hours and 44 hours post treatment, normalized to Veh (n=3). e. AMP levels measured by LC-MS of 143B cells treated with either Veh (DMSO) or 5 μM AA5 in DMEM extracted at 24 hours and 44 hours post treatment (n=3). f. Carbamoyl-aspartate (carb-Asp) levels measured by LC-MS of 143B cells treated with either Veh (DMSO) or 5 μM AA5 in DMEM extracted at 24 hours and 44 hours post treatment, normalized to Veh (n=3). g. UTP levels measured by LC-MS of 143B cells treated with either Veh (DMSO) or 5 μM AA5 in DMEM extracted at 24 hours and 44 hours post treatment normalized to Veh (n=3). h. GFP/RFP of 143B jAspSnFR3/NucRFP cells treated with either Veh (DMSO), 5 μM AA5, AA5 +500 μM Asn, AA5 +100 μM adenine (Ade), or AA5 +200 μM uridine (Uri) in DMEM (n=4). i. Aspartate levels measured by LC-MS in 143B cells treated with Veh (DMSO), 5 μM AA5, AA5 +200 μM Uri for 32 hours in DMEM, normalized to Veh (n=3). j. Carb-Asp levels measured by LC-MS in 143B cells treated with Veh (DMSO), 5 μM AA5, AA5 +200 μM Uri for 32 hours in DMEM, normalized to Veh (n=3). k. UTP levels measured by LC-MS in 143B cells treated with Veh (DMSO), 5 μM AA5, AA5 +200 μM Uri for 32 hours in DMEM, normalized to Veh (n=3). l. Model showing that uridine supplementation and incorporation causes feedback inhibition of *de novo* pyrimidine synthesis at the aspartate transcarbamoylase (ATCase) step and a depiction of how AA5 may impact ATCase activity through both impairing aspartate availability and a secondary effect that impairs ATCase function. Data are plotted as means ± standard deviation (SD) except h which is shown as means ± standard error of the mean (SEM). Statistics in this figure were generated using a two factor ANOVA with multiple comparisons and p-values for highlighted comparisons are shown above the horizontal lines on each plot.

We next sought to understand the aspartate rebound effect in SDH inhibited cells and its metabolic consequences. We first verified this conclusion, finding that treatment of 143B jAspSnFR3/NucRFP cells with AA5 once more caused an aspartate biosensor signal low-point around 24 hours and displayed a progressive restoration in aspartate signal over the ensuing 24 hours (Fig. 2a). To verify that these biosensor measurements were accurately reporting changes in aspartate abundance over time, we treated cells with either vehicle (Veh) or AA5 and extracted metabolites for LC-MS metabolomics at 24 hours, near the base of aspartate levels, or 44 hours, after aspartate levels were measured by jAspSnFR3 to have partially recovered. Indeed, aspartate was depleted by AA5 treatment at 24 hours and had partially recovered at 44 hours (Fig. 2b).

To explain this metabolic rebound in aspartate levels, we hypothesized that SDH inhibition causes secondary effects that impair aspartate utilization into its biosynthetic fates, impeding aspartate consumption and resulting in its accumulation. Aspartate supports biosynthesis through several mechanisms: 1) its direct role in tRNA charging for protein synthesis, 2) by serving as a precursor for the amino acids asparagine and arginine, and 3) by serving as a substrate to produce both purine and pyrimidine nucleotides (Fig. 2c). Therefore, we sought to measure how SDH inhibition impacts these metabolic fates. We first evaluated the effects of AA5 treatment on aspartate related tRNA charge levels, comparing the outcome of aspartate limitation from AA5 or rotenone treatment in WT cells or environmental aspartate limitation in GOT1/2 DKO cells. Notably, neither AA5 nor rotenone treatment caused consistent deficiencies in charging of aspartyl or asparaginyl tRNA species, suggesting tRNA charging deficiencies are unlikely to limit cell proliferation in these contexts (Extended Data Fig. 2a–b). Interestingly, GOT1/2 DKO cells did show tRNA charging deficiencies, particularly in mitochondrial aspartyl-tRNAs (Extended Data Fig. 2c–d). Nonetheless, while these results could indicate that these deficiencies may impact GOT1/2 DKO cell function during aspartate limitation, we note that cells experiencing these relatively substantial tRNA charging disruptions still maintained cell proliferation during modest aspartate limitation in this system, albeit slower (Extended Data Fig. 1g–h). Therefore, these findings indicate that deficiencies in tRNA charging are unlikely to be a major determinant of cell function upon AA5 treatment.

We also reanalyzed our LC-MS dataset to include other metabolic fates that are dependent on aspartate utilization, including asparagine, purine nucleotides, and metabolites in the pyrimidine synthesis pathway (Fig. 2c). Asparagine levels trended the same direction as aspartate, with the later timepoint of AA5 treated cells having higher levels than the earlier timepoint relative to vehicle treated cells, supporting the conclusion that aspartate levels are indeed elevated over time and that asparagine synthetase (ASNS) still operates in this context (Fig. 2d). AMP was not suppressed by AA5 at either timepoint, while GMP levels increased at both timepoints, perhaps due to the elevated NAD+/NADH ratio associated with AA5 treatment driving IMP to XMP, the precursor to the GMP synthetase reaction (Fig. 2e, Extended Data Fig. 3a)^12^. Interestingly however, the levels of the pyrimidine synthesis pathway intermediates carbamoyl-aspartate and dihydroorotate were impaired in AA5 treated cells and remained suppressed even after aspartate levels had partially rebounded at 44 hours (Fig. 2f, Extended Data Fig. 3b). The downstream metabolites UMP, UDP, and UTP in the pyrimidine biosynthesis pathway also largely remained similarly depleted at each time point (Fig. 2g, Extended Data Fig. 3c–d). Together, these data indicate that SDH inhibition causes an impairment to pyrimidine synthesis that exceeds what would be predicted from its effects on aspartate levels alone.

### Pyrimidine deficiency is required for the aspartate rebound in SDH inhibited cells

We next sought to investigate whether proximal impairments in aspartate utilization contributed to the rebound in aspartate levels in SDH impaired cells. Therefore, we sought methods to exogenously fulfill the major metabolic fates of aspartate and observe their consequences on aspartate levels. Since each potentially salvageable metabolic fate of aspartate has discrete uptake and incorporation characteristics, we first investigated the nutrient treatment conditions necessary to bypass the aspartate consumption demand for the synthesis of asparagine, pyrimidines, and purines. To do so, we used isotope tracing strategies to label endogenously produced aspartate fates and then determined the metabolite supplementation conditions that could displace the labeled species, indicating that *de novo* synthesis from aspartate was no longer necessary. As expected, treatment with unlabeled asparagine suppressed *de novo* asparagine synthesis and treatment with unlabeled uridine robustly suppressed the contribution of *de novo* pyrimidine synthesis to the UTP pool (Extended Data Fig. 4a, b). Isotope incorporation into arginine, indicative of *de novo* arginine synthesis, was undetectable in standard conditions, consistent with standard media conditions containing an excess of unlabeled arginine that suppresses arginine production and with prior findings that many cancer cell lines suppress *de novo* arginine synthesis pathways (Extended Data Fig. 4c)^25,26^. Among purine nucleobase treatments, adenine was sufficient to meet all purine demands. It not only bypassed the aspartate consumption step that is specific to adenine nucleotide synthesis but also supported guanine nucleotide production, presumably through deamination to generate hypoxanthine/IMP, circumventing the earlier aspartate consumption step common to all *de novo* purine nucleotide synthesis (Fig. 2c, Extended Data Fig. 4d). Altogether, these results identify that 100 μM adenine, 200 μM uridine, and 500 μM asparagine can efficiently fulfill the non-protein metabolic fates of aspartate in these systems.

We then evaluated how supplementation of aspartate fates impacted the dynamic effects of SDH inhibition on aspartate levels. Strikingly, while treatment with adenine or asparagine did not substantially affect aspartate kinetics in AA5 treated cells, supplementation with uridine abolished the aspartate rebound and resulted in monotonic aspartate depletion (Fig. 2h). Interestingly, despite these large effects on aspartate homeostasis, supplementing with these individual fates only had modest and temporary effects to improve the proliferation rate of SDH impaired 143B cells (Extended Data Fig. 5a–b). Confirming that this effect was specific to SDH inhibition, we found that rotenone treated 143B cells showed no differences in aspartate levels or proliferation rate upon uridine supplementation (Extended Data Fig. 5c–d). The rebound of aspartate levels in SDH impaired cells was also observed in jAspSnFR3/NucRFP expressing H1299 and HCT116 cells and, in both cases, was also diminished by uridine co-treatment (Extended Data Fig. 5e–h). LC-MS metabolomics measurements 32 hours post-treatment confirmed that uridine supplementation indeed depleted aspartate levels in SDH-impaired 143B cells, in agreement with biosensor readouts (Fig. 2i). Consistent with known feedback regulation, salvaged pyrimidines also suppressed carbamoyl-aspartate synthesis, as uridine nucleotides are known to negatively regulate carbamoyl-aspartate synthesis by impairing the aspartate transcarbamoylase (ATCase) activity of the trifunctional enzyme carbamoyl-phosphate synthetase 2, aspartate transcarbamoylase, and dihydroorotase (CAD) (Fig. 2j)^27^. Indeed, uridine supplementation fully restored UTP levels in SDH-impaired cells (Fig. 2k). These results are therefore consistent with a model where a consequence of SDH inhibition is a secondary impairment of aspartate utilization into pyrimidine synthesis at the ATCase step, resulting in slowed aspartate consumption and a rebound in aspartate levels over time (Fig. 2l).

### Quantification of aspartate contributions to metabolic fates

One striking finding of these experiments is that despite the robust effect of uridine supplementation on aspartate homeostasis, the benefits to cell proliferation in AA5 treated cells were surprisingly modest (Extended Data Fig. 5a–b, f–h). Rotenone treated 143B cells similarly did not gain a substantial proliferation benefit from uridine supplementation (Extended Data Fig. 5d). To contextualize how aspartate fate supplementation might impact the demand on aspartate consumption, we sought to quantify the aspartate demands to support each of its metabolic fates during cell proliferation. To estimate the total rate of aspartate usage and incorporation into each fate, we measured amino acid uptake and release from GOT1/2 DKO 143B cells expressing the aspartate transporter SLC1A3, which provided a model to quantify total aspartate flux without synthesis. In general, we observed consumption patterns of amino acids that match prior findings, and the release of some amino acids absent in DMEM, such as asparagine and proline, as expected (Extended Data Fig. 6a)^28^. Notably, aspartate exhibited the second fastest amino acid uptake in these cells at approximately 11 mM/h, consistent with high demand to fulfill several metabolic fates.

We next sought to determine the flux of aspartate in wild type (WT) cells. First, we measured the flux of asparagine in WT 143B cells, by conducting uptake/release measurements in the presence of U-^13^C asparagine and measuring final intracellular asparagine labeling, which enabled calculations of asparagine flux into protein and efflux (Extended Data Fig. 6b–d, see Methods). We note that the calculated rate of asparagine efflux in WT cells closely aligned with the measured rate in GOT1/2 DKO cells without asparagine supplementation (Extended Data Fig 6a, d). Next, we conducted acid hydrolysis of 143B and H1299 cells to quantify total amino acid and nucleotide composition, including both free metabolites and their liberated versions from protein and RNA/DNA (Extended Data Fig. 6e). Since acid hydrolysis converts glutamine to glutamate and asparagine to aspartate, these variables are measured as combined concentrations. Purines are also partially destroyed by acid hydrolysis, so their levels were estimated to match pyrimidines based on prior studies^29,30^. Total cell concentrations and proliferation rate measurements were converted into flux, with aspartate’s contribution to protein inferred by subtracting the calculated asparagine-to-protein flux from the total aspartate/asparagine flux (Extended Data Fig. 6d, f). Flux calculations indicated that protein synthesis is the primary consumer of aspartate, while asparagine efflux also serves as a significant sink, influenced by media composition and likely as a transient term as the media becomes conditioned by asparagine leakage. Excluding arginine flux, which is not synthesized by cells in DMEM (Extended Data Fig. 4c), we estimated a total aspartate demand of ~10 mM/h in 143B cells, closely matching the measured uptake in GOT1/2 DKO 143B cells (Extended Data Fig. 6a, f).

Together, these results demonstrate that aspartate has high and distributed demands to fulfill its metabolic fates. Notably, no individual fate can obviate a majority of aspartate demands, it is therefore reasonable that sparing aspartate consumption into pyrimidine synthesis would only provide an incremental proliferation benefit, as is also true for each aspartate fate, individually (Extended Data Fig. 5a, 6f)^23,31,32^. These findings indicate that the rise in aspartate levels in SDH impaired cells signifies a temporary imbalance of aspartate synthesis and consumption resulting from a superseding pyrimidine synthesis impairment that ultimately resolves once aspartate levels rebound, with only a small net effect on cell proliferation rate when measured over multiple days.

### Succinate competitively inhibits aspartate utilization into pyrimidines at aspartate transcarbamoylase

We next sought to understand the mechanisms underlying the complex effects of SDH inhibition on aspartate utilization and pyrimidine synthesis. The impairment in pyrimidine synthesis appears to occur at ATCase, as its product, carbamoyl-aspartate—the first committed metabolite in pyrimidine synthesis—is depleted along with its downstream fates. A key metabolic distinction between SDH inhibition and other causes of aspartate limitation is the accumulation of succinate, the substrate of SDH. Succinate is known to have multiple biochemical effects, including serving as a competitive inhibitor of various alpha ketoglutarate (αKG) dependent dioxygenases involved in oxygen sensing and DNA/histone demethylation^33^. Interestingly, the ATCase activity of *Escherichia Coli* CAD has been a workhorse experimental model for understanding enzyme kinetics for over a half century and prior work on purified bacterial CAD enzymes has found that, at high concentrations, succinate can competitively inhibit aspartate utilization by ATCase^34–37^. To our knowledge, succinate inhibition of ATCase has not been described for mammalian ATCase, nor has this interaction been observed within the bounds of metabolite levels in a living cell system. Nonetheless, the human ATCase domain of CAD has a high degree of structural similarly to its bacterial homologs, including nearly identical active sites, supporting the plausibility that human ATCase would be similarly affected by high succinate levels^38^. We thus hypothesized that accumulated succinate impairs ATCase activity in SDH-inhibited cells, which is compounded by aspartate depletion, and sought to evaluate this hypothesis by testing whether modulating the levels of aspartate and succinate in SDH-impaired cells could correspondingly impact aspartate levels and pyrimidine synthesis.

**Figure 3.**
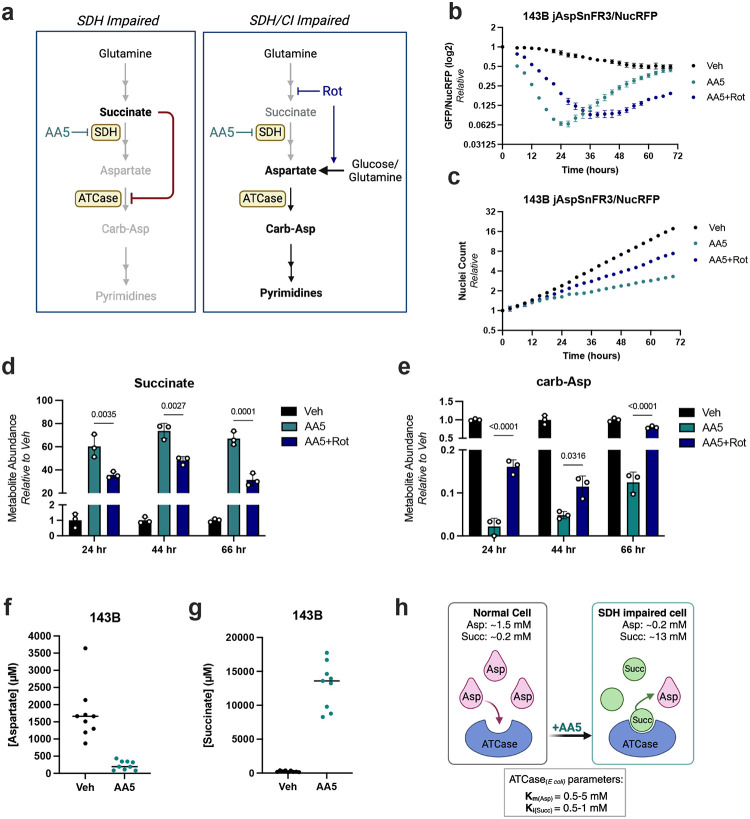
Decreasing succinate levels disinhibits aspartate utilization into pyrimidines at aspartate transcarbamoylase a. Model depicting the hypothesis that accumulated succinate from SDH inhibition (AA5) competitively inhibits aspartate consumption at aspartate transcarbamoylase (ATCase) to impair *de novo* pyrimidine synthesis. The addition of complex I inhibition by rotenone (Rot) treatment increases alternative aspartate synthesis pathways and decreases succinate synthesis, lowering succinate levels. These dual effects could then disinhibit ATCase and restore *de novo* pyrimidine synthesis. b. GFP/RFP of 143B jAspSnFR3/NucRFP cells treated with either Veh (DMSO), 5 μM AA5, or 5 μM AA5 +50 nM rotenone (Rot) in DMEM (n=3). c. NucRFP counts of 143B jAspSnFR3/NucRFP cells treated with either Veh (DMSO), 5 μM AA5, or 5 μM AA5 +50 nM rotenone in DMEM (n=3). d. Relative succinate levels measured by LC-MS in 143B cells treated with either Veh (DMSO), 5 μM AA5, or 5 μM AA5 +50 nM Rotenone in DMEM at 24, 44, and 66 hours post treatment (n=3). e. Relative carbamoyl-aspartate (carb-Asp) levels measured by LC-MS in 143B cells treated with either Veh (DMSO), 5 μM AA5, or 5 μM AA5 +50 nM rotenone in DMEM 24, 44, and 66 hours post treatment (n=3). f. Intracellular aspartate concentrations measured by absolute quantification by LC-MS in 143B cells treated with either Veh (DMSO) or 5 μM AA5 in DMEM taken from several experiments with metabolites collected up to 50 hours post-treatment (n=9). g. Intracellular succinate concentrations measured by absolute quantification by LC-MS in 143B cells treated with either Veh (DMSO) or 5 μM AA5 in DMEM taken from several experiments with metabolites collected up to 50 hours post-treatment (n=9). h. Model defining enzyme substrate parameters for aspartate transcarbamoylase (ATCase) in normal vs. SDH impaired cells. For *E. coli* ATCase, previous studies have found a K_m_ for aspartate of 0.5–5 mM and a K_*i*_ for succinate of 0.5–1 mM. Median aspartate and succinate concentration measurements from 143B cells at baseline are around 1.5 mM for aspartate and 200 μM for succinate, which would not be predicted to impair ATCase function. In SDH impaired cells, the measured values around 200 μM for aspartate and 13 mM for succinate are predicted to inhibit ATCase, assuming similar biochemical parameters for human ATCase. Data are plotted as means ± standard deviation (SD). Statistics in this figure were generated using a two-way ANOVA with multiple comparisons and p-values for highlighted comparisons are shown above the horizontal lines on each plot.

We recently reported that SDH inhibited cells benefit from co-inhibition of ETC complex I (CI), which improves cell proliferation by decreasing mitochondrial NAD+/NADH to promote alternative aspartate synthesis pathways^12^. In addition to increasing aspartate production, complex I inhibition is also predicted to decrease succinate accumulation by slowing the activity of the NAD+ dependent mitochondrial enzyme alpha-ketoglutarate dehydrogenase (αKGDH)^12,14^, thus potentially having dual impacts on aspartate homeostasis by increasing aspartate synthesis while disinhibiting aspartate consumption by ATCase. Indeed, live cell imaging of jAspSnFR3/NucRFP 143B cells found that co-treatment with the complex I inhibitor rotenone delayed the initial decline in aspartate levels in AA5 treated cells and attenuated the aspartate rebound effect (Fig. 3b). Strikingly, these changes in aspartate dynamics resulted in late time points where steady state aspartate levels were lower in the faster proliferating cells co-treated with rotenone, compared to those treated with AA5 alone (Fig. 3b–c), even though aspartate supplementation promotes proliferation in the latter condition (Fig. 1i)^12^.

We next investigated the effects of inhibition of SDH or SDH/CI on metabolite levels over time by LC-MS. Corroborating the aspartate biosensor measurements, aspartate levels were more depleted in cells treated with AA5 alone than cells co-treated with rotenone at early time points, a relationship that had flipped by 44 hours (Extended Data Fig. 7a). As expected, SDH inhibition also substantially increased succinate levels, which were partially mitigated by CI co-inhibition with rotenone, a relationship that was stable over time (Fig. 3d). Importantly, SDH/CI co-inhibition also correspondingly increased the levels of carbamoyl-aspartate at each timepoint relative to those with SDH inhibition alone (Fig. 3e). Together these data corroborate the hypothesis that succinate competes with aspartate utilization at ATCase to determine the pyrimidine synthesis rate and govern the aspartate rebound effect in SDH impaired cells.

To evaluate whether the biochemical parameters present in SDH impaired cells were consistent with a competitive interaction at ATCase, we sought to understand the range of aspartate and succinate concentrations in cells with or without SDH inhibition. We thus conducted absolute quantification for aspartate and succinate by LC-MS from cells treated with vehicle or AA5, combining concentrations from multiple experiments and time points. We found that vehicle treated 143B cells maintain median aspartate levels around 1.7 mM and succinate levels around 200 μM, consistent with other metabolite quantification measurements of unperturbed mammalian cells (Fig. 3f–g)^2,39^. SDH inhibition precipitously decreased aspartate concentrations, with some variability depending on time point, to a median concentration of approximately 200 μM (Fig. 3f). SDH inhibition also increased succinate levels by approximately 70-fold, reaching a median concentration of around 14 mM, which is comparable to what has been measured in SDH deficient tumors (Fig. 3g)^40–42^. While *in vivo* CAD activity is likely strongly influenced by other factors such as signaling state, allosteric effectors, and feedback inhibition, the finding that intracellular concentrations of aspartate and succinate are well within the biochemical range to impair ATCase function thus supports the concept that the combination of aspartate depletion and succinate accumulation can collaboratively impair ATCase function in these contexts (Fig. 3h)^35,36,43–45^.

To further evaluate whether CI inhibition restores pyrimidine synthesis to SDH impaired cells, we conducted U-^13^C glutamine tracing in cells treated with vehicle, AA5 alone, or AA5+Rot after 32 hours of treatment, which previous measurements showed is approximately when aspartate levels from the two treatments overlap (Fig. 3b, Extended Data Fig. 7a). Indeed, aspartate levels were near equivalent between the AA5 and AA5+Rot conditions at this time point, although the isotopologue distribution was shifted towards increased M+3 in the AA5+Rot condition, consistent with prior observations that CI inhibition activates reductive carboxylation of glutamine-derived αKG (Extended Data Fig. 7b)^12,46^. Notably, while both AA5 treated conditions had near identical aspartate abundance, rotenone co-treated cells had decreased succinate levels and substantially restored pyrimidine synthesis pathway intermediates (Extended Data Fig. 7c–g). These metabolites also displayed appropriate ^13^C labeling patterns demonstrating that these pyrimidine intermediates were derived from biosynthesis, further indicating that the pathway had been disinhibited by the effects of rotenone on lowering succinate levels (Extended Data Fig. 7c–g).

### Pyrimidine synthesis defects are not a generalized consequence of acute TCA cycle dysfunction

Our data indicate that the effects of SDH inhibition cause dynamic changes to many metabolic variables and cell proliferation, raising the possibility that observed consequences on pyrimidine synthesis are influenced by other acute effects of TCA cycle dysfunction. To address this possibility, we sought a system where TCA cycle status was constant across conditions. Hence, we used CRISPR/Cas9 to generate monoclonal cells deficient in fumarate hydratase (FH), the metabolic enzyme downstream of SDH that converts fumarate to malate, in 143B cells (Extended Data Fig. 8a). FH knockout causes cells to accumulate fumarate and can partially accumulate succinate in cells and tumors, although not nearly to the degree observed in cells with SDH deficiency^40,47,48^. We thus tested if SDH inhibition by AA5 could further accumulate succinate in FH KO cells and thereby provide an opportunity to evaluate the effects of succinate accumulation without drastic changes to the bioenergetic state of these cells. We treated FH KO 143B cells with AA5 and conducted metabolomics at 48 hours. As expected, we measured a substantial shift from fumarate accumulation to succinate accumulation upon AA5 treatment (Extended Data Fig. 8b–c). Strikingly, despite equivalent impairments to TCA cycle functionality, SDH inhibition increased aspartate levels in FH KO cells while decreasing carbamoyl-aspartate and downstream pyrimidine nucleotides, mirroring the effects of succinate accumulation observed in cells with otherwise intact TCA cycle function upon treatment with AA5 (Extended Data Fig. 8d–g). Notably, this effect was specific to impairing aspartate utilization in pyrimidine synthesis, since asparagine levels still tracked with increased aspartate and AMP levels remain constant, as previously observed (Extended Data Fig. 8h–i). These data confirm that the bioenergetic effects of blocking the oxidative TCA cycle is not sufficient on its own to cause pyrimidine synthesis inhibition; rather, this effect requires accumulated succinate from SDH inhibition.

### High succinate drives pyrimidine synthesis inhibition

**Figure 4.**
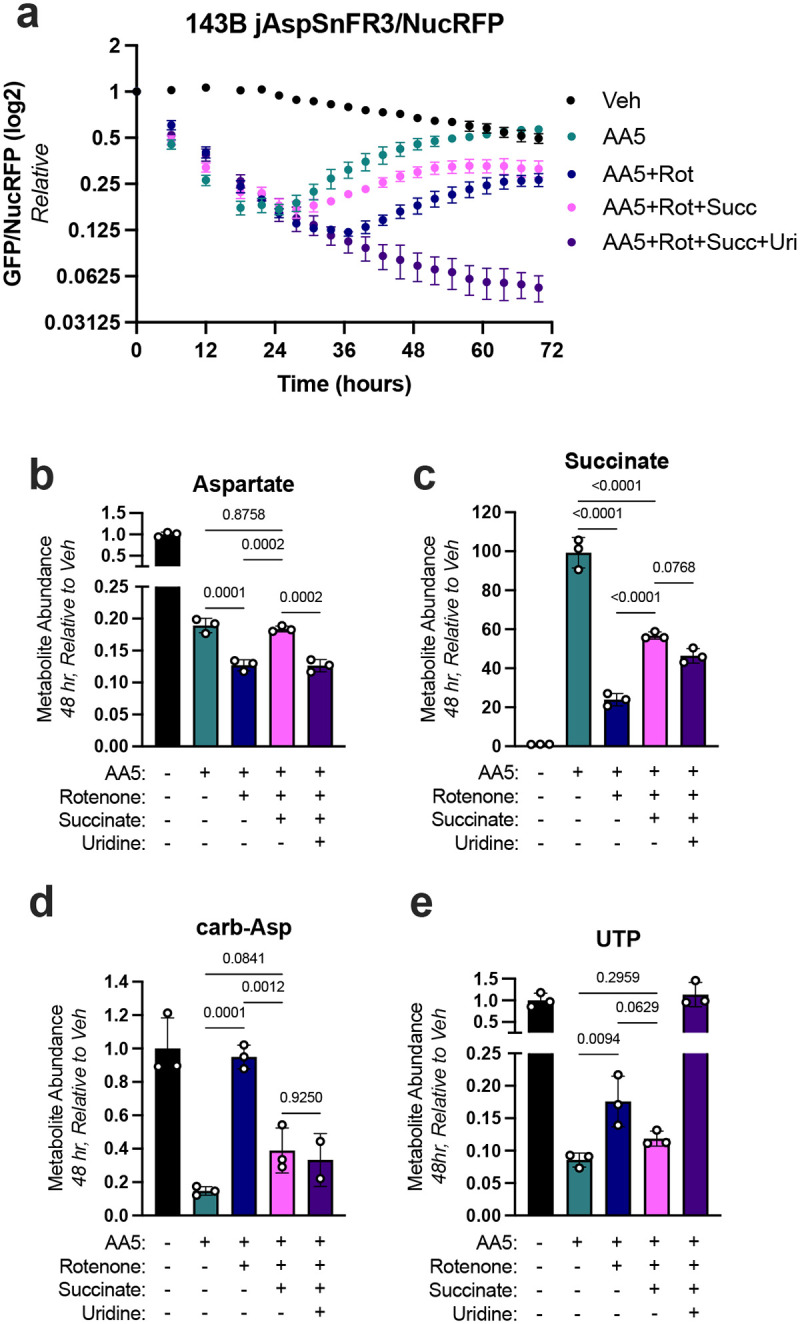
Succinate accumulation is sufficient to impair aspartate utilization into pyrimidines at aspartate transcarbamoylase a. GFP/RFP of 143B jAspSnFR3/NucRFP cells treated with either Veh (DMSO), 5 μM AA5, 5 μM AA5 +50 nM rotenone (Rot), 5 μM AA5 +50 nM Rot +10 mM succinate (Succ), or 5 μM AA5 +50 nM Rot +10 mM succinate +200 μM uridine (Uri) in DMEM (n=3). b. Aspartate levels of 143B cells measured by LC-MS treated with either Veh (DMSO), 5 μM AA5, 5 μM AA5 +50 nM Rot, 5 μM AA5 +50 nM Rot +10 mM Succ, or 5 μM AA5 +50 nM Rot +10 mM Succ +200 μM Uri (n=3) for 48 hours in DMEM, normalized to Veh (n=3). c. Succinate levels of 143B cells measured by LC-MS treated with either Veh (DMSO), 5 μM AA5, 5 μM AA5 +50 nM Rot, 5 μM AA5 +50 nM Rot +10 mM Succ, or 5 μM AA5 +50 nM Rot +10 mM Succ +200 μM Uri (n=3) for 48 hours in DMEM, normalized to Veh (n=3). d. Carb-Asp levels of 143B cells measured by LC-MS treated with either Veh (DMSO), 5 μM AA5, 5 μM AA5 +50 nM Rot, 5 μM AA5 +50 nM Rot +10 mM Succ, or 5 μM AA5 +50 nM Rot +10 mM Succ +200 μM Uri (n=3) for 48 hours in DMEM, normalized to Veh (n=3). e. UTP levels of 143B cells measured by LC-MS treated with either Veh (DMSO), 5 μM AA5, 5 μM AA5 +50 nM Rot, 5 μM AA5 +50 nM Rot +10 mM Succ, or 5 μM AA5 +50 nM Rot +10 mM Succ +200 μM Uri (n=3) for 48 hours in DMEM, normalized to Veh (n=3). Data are plotted as means ± standard deviation (SD). Statistics in this figure were generated using an ordinary one-way ANOVA test with multiple comparisons and p-values for highlighted comparisons are shown above the horizontal lines on each plot.

Next, we queried whether succinate supplementation would be sufficient to impair pyrimidine synthesis and correspondingly promote a rebound effect in aspartate levels. One challenge with this goal is that providing succinate to cells with intact SDH activity could be counterproductive, since the acquired succinate would be metabolized by SDH, diminishing the intended effect and likely promoting the production of downstream metabolites, including aspartate. We thus sought to evaluate how succinate supplementation impacts aspartate and pyrimidine synthesis in cells without functional SDH, but also without the full aspartate rebound already present. We thus leveraged the condition of cells co-treated with AA5 and rotenone, which decreases endogenous succinate synthesis and mitigates the aspartate rebound effect (Fig. 3b). Strikingly, we found that succinate treatment was sufficient to restore the aspartate rebound effect in cells co-treated with AA5 and rotenone (Fig. 4a). To test whether this effect was also due to pyrimidine insufficiency, we co-treated these cells with uridine and found that the aspartate rebound was again abolished (Fig. 4a). To validate that succinate was entering cells and impairing pyrimidine biosynthesis in SDH/CI impaired cells, we conducted metabolomics at 48 hours post treatment. We confirmed that both succinate and aspartate levels were elevated post-rebound in SDH/CI impaired cells when succinate was provided, while carbamoyl-aspartate and UTP were once again suppressed (Fig. 4b–e). Collectively, these results further demonstrate that succinate accumulation from SDH inhibition competitively impairs *de novo* pyrimidine synthesis at the aspartate-incorporating ATCase reaction of CAD.

Altogether, these data suggest a model to describe the aspartate rebound phenomenon in SDH deficient cells, in several phases: 1) SDH inhibition lowers aspartate production while proliferation rate is initially maintained, decreasing aspartate levels; 2) Aspartate depletion slows proliferation, steadying the loss of aspartate levels; 3) Accumulated succinate competitively inhibits ATCase, compounded by decreased aspartate substrate availability, impairing *de novo* pyrimidine synthesis and partially limiting aspartate consumption from cell proliferation, resulting in aspartate accumulation; 4) Aspartate accumulates until it reaches a concentration that can overcome succinate inhibition at ATCase, leading to a new pseudo-steady state of matched aspartate production and consumption rates.

### SDH inhibition causes replication stress through impairments to pyrimidine synthesis

**Figure 5.**
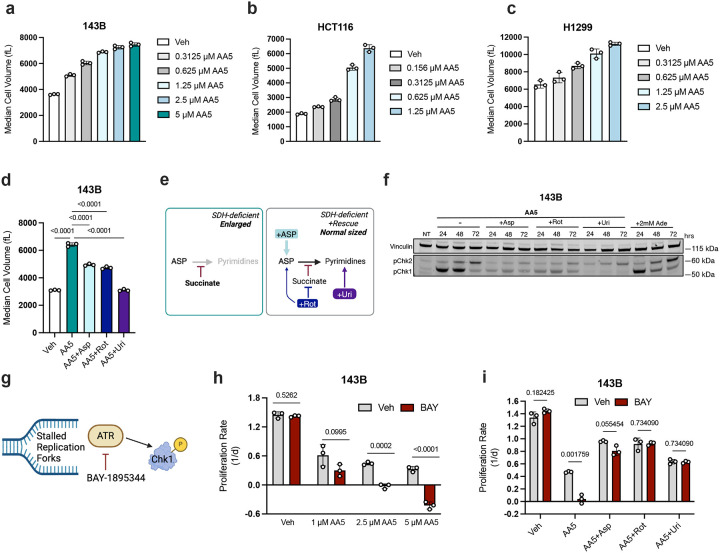
Pyrimidine synthesis inhibition activates replication stress in SDH impaired cells a. Median cell volumes of 143B cells treated for 96 hours with an AA5 titration in DMEM (n=3). b. Median cell volumes of H1299 cells treated for 72 hours with an AA5 titration in DMEM (n=3). c. Median cell volumes of HCT116 cells treated for 72 hours with an AA5 titration in DMEM (n=3). d. Median cell volumes of 143B cells treated for 72 hours with either DMSO, 5 μM AA5, 5 μM AA5 +20 mM aspartate, 5 μM AA5 +50 nM rotenone, or 5 μM AA5 +200 μM uridine in DMEM (n=3). e. Model characterizing the pyrimidine synthesis impairment in SDH deficient cells and associated enlargement phenotype, which results from aspartate depletion and succinate accumulation. Methods to restore pyrimidine synthesis to SDH deficient cells by 1) Direct aspartate treatment to restore aspartate levels, outcompeting the effects of succinate accumulation; 2) Rotenone co-treatment, increasing aspartate production while decreasing succinate accumulation; or 3) Direct supplementation of uridine, all prevent the cell enlargement phenotype. f. Western blot measuring the expression of phosphorylated Chk1, phosphorylated Chk2, and vinculin (loading control) in 143B cells treated for the indicated time with either no treatment (NT, treated with vehicle (DMSO)), 5 μM AA5, 5 μM AA5 +20 mM aspartate (+Asp), 5 μM AA5 +50 nM rotenone (+Rot), 5 μM AA5 +200 μM uridine (+Uri), or 2 mM adenine as a positive control for nucleotide imbalance initiated replication stress in DMEM. g. ATR kinase responds to replication stress resulting from stalled replication forks by phosphorylating Chk1. BAY-1895344 is a specific inhibitor of ATR. h. Proliferation rates of 143B cells after 72 hours of treatment with the indicated dose of AA5 with or without 20 nM BAY-1895344 (BAY) in DMEM (n=3). i. Proliferation rates of 143B cells after 72 hours of treatment with either Veh (DMSO), 5 μM AA5, 5 μM AA5 +20 mM aspartate, 5 μM AA5 +50 nM rotenone, or 5 μM AA5 +200 μM uridine with or without 20 nM BAY-1895344 (BAY) in DMEM (n=3). Data are plotted as means ± standard deviation (SD). Statistics in this figure were generated using multiple paired student’s t-tests and p-values for highlighted comparisons are shown above the horizontal lines on each plot.

Imbalanced nucleotide availability can induce activation and dependence on replication stress signaling^49,50^. Cells experiencing replication stress are defined by issues with DNA replication, resulting in a prolonged S phase, phosphorylation of Chk1 and Chk2 by ATR and/or ATM, H2A.X phosphorylation, and impairments to cell proliferation^51–54^. In cancer cells, these cell proliferation defects can occur without commensurate decreases in growth signaling or protein synthesis, resulting in larger cells^49^. We thus tested whether the secondary impairments to pyrimidine synthesis that occur from SDH inhibition could similarly promote replication stress phenotypes. Indeed, we noted that treatment with AA5 caused a dose-dependent increase in cell volume, commensurate with dose-dependent decreases in cell proliferation in several cell lines (Fig. 5a–c, Extended Data Fig. 9a–c). When causing aspartate limitation in 143B cells through other perturbations - rotenone treatment in pyruvate free media or low media aspartate GOT1/2 DKO cells - we also noted a modest increase in cell volume in the most aspartate limited conditions (Extended Data Fig. 9d–e). However, in both cases, the magnitude of effect was much smaller than that caused by AA5 treatment, indicating that SDH inhibition caused a more substantial replication stress phenotype than these other modes of aspartate limitation. We also observed that FH KO 143B cells are not inherently larger than WT 143B cells, demonstrating that this phenotype is not inherently a result of TCA cycle dysfunction (Extended Data Fig. 9f). Thus, these data indicate that the robust cell enlargement phenotype upon SDH inhibition stems from its specific effects on pyrimidine synthesis blockade, rather than generalized aspartate depletion or TCA cycle dysfunction. Indeed, cell enlargement from SDH inhibition is partially reversed by aspartate or rotenone treatment and is fully rescued by uridine supplementation in several cell lines (Fig. 5d, Extended Data Fig. 9g–h). Similarly, AA5 treatment also increased the size of FH KO cells, which was similarly rescued by treatment with aspartate or uridine (Extended Data Fig. 9i). Interestingly, each of these rescue treatments has drastically different effects on aspartate levels^12^ (Fig. 2h, 3b, Extended Data Fig. 1o), but each converges by providing a mechanism to restore pyrimidine nucleotides (Fig. 5e). We also queried whether exogenous succinate treatment would cause SDH/CI impaired cells to swell, and found a dose-dependent effect, while exogenous succinate had no effect on untreated cell volume (Extended Data Fig. 9j).

To directly evaluate replication stress signaling, we measured phosphorylation of Chk1 and Chk2 at 24, 48, and 72 hours post AA5 treatment, with or without treatments to restore pyrimidine synthesis. SDH inhibition first promoted Chk1 phosphorylation, followed by Chk2 phosphorylation, consistent with the ATR activated replication stress signaling observed in other contexts of nucleotide imbalance (Fig. 5f)^49^. Indeed, supraphysiological adenine treatment, which has been shown to cause ATR dependent replication stress, also caused similar phosphorylation events (Fig. 5f)^49^. Importantly, additional treatment with aspartate, rotenone, or uridine mitigated Chk1/2 phosphorylation commensurate with their ability to prevent cell enlargement (Fig. 5d, f). In H1299 cells, rotenone failed to prevent cell enlargement of AA5 treated cells or Chk1 phosphorylation, indicating that these cells may experience different metabolic effects from complex I co-inhibition (Extended Data Fig. 9h, k). This activation of Chk1 phosphorylation by AA5 was also a result of ATR signaling, since treatment with either of the ATR specific inhibitors BAY-1895344 (BAY) or VE-821 suppressed Chk1 phosphorylation without affecting Chk2 phosphorylation, as expected (Extended data Fig. 9l)^55,56^. Together, these data highlight that SDH inhibition is sufficient to cause ATR-dependent replication stress signaling, resulting from nucleotide imbalances caused by pyrimidine synthesis inhibition.

ATR signaling is essential during nucleotide imbalance to prevent a catastrophic loss in cell proliferative capacity from cell death, quiescence, or senescence (Fig. 5g)^57^. We thus tested whether SDH impaired cells would exhibit increased vulnerability to ATR inhibition. Indeed, while the ATR inhibitor BAY had no effect on untreated cells, BAY treatment synergized with AA5 to abolish cell proliferation in multiple cell lines (Fig. 5h, Extended Data 9m–o). Notably, this sensitivity was not merely a result of the aspartate limitation from SDH inhibition slowing cell proliferation, since cells that were comparably slowed by rotenone treatment in pyruvate free media were unaffected by BAY treatment (Extended Data Fig. 9p). Instead, our data indicate that this synergy in SDH-deficient cells was dependent on the secondary impairments to pyrimidine synthesis, since aspartate, rotenone, or uridine treatment were all sufficient to abolish the toxicity of BAY and VE-821 in AA5 treated cells (Fig. 5i, Extended Data Fig. 9q–r). Altogether, these results provide evidence that replication stress signaling is activated in SDH-deficient cells, caused by pyrimidine synthesis impairments from high succinate and decreased aspartate, and is resolved by any means to restore pyrimidine nucleotides.

## Discussion:

Here, we leverage an aspartate biosensor and live cell imaging to temporally resolve how conditions that constrain aspartate acquisition impact cell metabolism and function. Interestingly, we observe that these metabolic perturbations do not immediately cause functional defects since hours of aspartate depletion precede their effects on cell proliferation. These results further highlight the relevance of aspartate depletion to mediating functional effects in these settings and demonstrate how useful insights can be gleaned from understanding the kinetics of cell proliferation changes. Temporal measurements of aspartate levels in SDH impaired cells also captured a surprising phenomenon where aspartate levels decline and then rebound over time. Notably, this capricious effect was so substantial that, when comparing the effects of SDH inhibition with or without co-treatment with a complex I inhibitor, the condition with the higher relative aspartate levels switches depending on the time point chosen, providing a striking example of how the dynamic nature of metabolic changes may be missed when measuring limited timepoints. These findings therefore underscore the importance of technology development that enables robust measurements of cell function and metabolism over time.

Our work contributes to our growing understanding of how metabolites can regulate cell function beyond their roles as direct catabolic or anabolic substrates. Indeed, we find that accumulated succinate can serve as a competitive inhibitor of aspartate utilization for the ATCase activity of CAD, therefore impairing the first committed step of *de novo* pyrimidine biosynthesis. This mechanism therefore adds another regulatory function of succinate, which has also been described to promote hypoxic signaling responses, modify histone and DNA methylation states, and activate signaling cascades through Succinate Receptor 1 (SUCNR1)^58^. Notably, since succinate accumulation can drive biological phenotypes in diverse contexts, including SDH-deficient cancers, ischemia/reperfusion, liver inflammation, macrophage function, and brown adipose tissue thermogenesis, it will be important to consider how changes in pyrimidine synthesis may influence the biological phenotypes of succinate accumulation in each context^58–66^.

This study reinforces the principle that metabolite levels can neither directly equate to pathway flux nor inherently be used to identify metabolic limitations - common misconceptions in metabolism research^67^. Metabolite levels represent the balance between production and consumption, and so identical effects on metabolite levels can arise from opposite changes to its metabolic fluxes (e.g. increased synthesis or decreased consumption can both cause metabolite accumulation)^68^. When evaluating how metabolite level changes impact cell function during metabolic perturbations, a straightforward intuition is that depleted metabolites may represent metabolic limitations and that their restoration would commensurately rescue cell function. Indeed, our results shown that this simple model holds for aspartate depletion during complex I inhibition or aspartate withdrawal in GOT1/2 DKO cells. However, our findings during SDH inhibition add another layer of complexity to this assumption, since we describe a context where aspartate is a metabolic limitation for cell proliferation even when it is relatively accumulated compared to other conditions due to competitive interactions from contemporaneous metabolite level changes. Interestingly, this phenotype is not unique to SDH loss, since aspartate has also been described to be functionally limiting for cell proliferation in CD8+ T cells experiencing iron deficiency, even while those cells accumulate aspartate^69^. Collectively, the findings further demonstrate the need for holistic understanding of metabolic fluxes and cell behaviors to conclusively link metabolomic alterations with functional phenotypes.

Lastly, we describe a phenotype that separates SDH inhibition from other causes of aspartate limitation through its disproportionate impairment of pyrimidine synthesis. We observe that the corresponding depletion of pyrimidine intermediates from SDH inhibition causes DNA replication stress, a phenotype well described in other contexts of nucleotide imbalance^49,50,70,71^. Congruently, we find that SDH impaired cells activate replication stress response pathways, including ATR-dependent Chk1 phosphorylation, and are exquisitely dependent on ATR function to maintain cell survival and proliferation. Considering that members of the SDH complex are also tumor suppressors in renal cell carcinoma and several neuroendocrine cancers, these findings also raise the question of whether SDH-mutant tumors may similarly endure replication stress. Intriguingly, cancer cells and tumors with loss of function mutations in the SDH complex have been found to have constitutively elevated phospho-H2A.X, another consequence of replication stress^40,72,73^. Furthermore, transcriptomic profiling of a mouse model of SDH-deficient pheochromocytoma found increased expression of DNA repair pathways and pyrimidine metabolism genes, suggesting similar metabolic stressors^74^. Our findings therefore raise new possibilities for approaches to target SDH-mutant cancers by leveraging unique synthetic lethalities that arise from the impact of SDH loss on pyrimidine synthesis.

## Materials and Methods:

### Cell Culture

Cell lines were acquired from ATCC (143B, H1299, HCT116) and tested to be free from mycoplasma (MycoProbe, R&D Systems). Cells were maintained in Dulbecco’s Modified Eagle’s Medium (DMEM) (Gibco, 50–003-PB) supplemented with 3.7 g/L sodium bicarbonate (Sigma-Aldrich, S6297), 10% fetal bovine serum (FBS) (Gibco, 26140079) and 1% penicillin-streptomycin solution (Sigma-Aldrich, P4333). Cells were incubated in a humidified incubator at 37°C with 5% CO_2_.

### Generation of jAspSnFR3/NucRFP cell lines

Nuclear RFP cell lines were generated as previously described^2^. jAspSnFR3 lentivirus was generated by co-transfection of HEK293T cells with p-Lenti-jAspSnFR3 (Addgene, 203488) and the packaging plasmids pMDLg/pRRE (Addgene, 12251), pRSV-Rev, (Addgene, 12253) and pMD2.G (Addgene, 12259) using FuGENE transfection reagent (Fisher, PRE2693) in DMEM (Fisher, MT10017CV) without FBS or penicillin-streptomycin. The supernatant containing lentiviral particles was filtered through a 0.45 μM membrane (Fisher, 9720514) and was supplemented with 8 μg/μL polybrene (Sigma, TR-1003-G) prior to infection. For infection, 143B, GOT1/2 DKO 143B, and HCT116 cells were seeded at 50% confluency in 6 well dishes and centrifuged with lentivirus (900g, 90 mins, 30°C). After 24 hours the media was replaced with fresh media and after 48 hours cells were treated with 150 μg/mL hygromycin (Sigma-Aldrich, H7772–1G) and maintained in selection media until all uninfected control cells died. After selection, cells were expanded and single cell cloned by limiting dilution, plating 0.5 cells/well using two 96 well plates. These clones were incubated until 10–30% confluency and screened for high GFP and RFP signal using an Incucyte S3 (Sartorius). The highest expressing monoclonal cells were selected and further expanded on 6 well plates and again screened for high fluorescence using the Incucyte. From this, single clones were chosen, expanded and used for all subsequent experiments. H1299 and GOT1/2 DKO H1299 cells already expressed jAspSnFR3 and nuclear RFP^2^ where WT 143B, GOT DKO 143B, and HCT116 cells were engineered to express nuclear RFP (Cellomics, PLV-10205–50) and pLenti-jAspSnFR3 for this manuscript. GOT1/2 DKO 143B and H1299 cells (no aspartate sensor) with and without SLC1A3 expression were also previously generated^2^.

### jAspSnFR3 and NucRFP Incucyte measurements

Experiments were conducted in either DMEM without pyruvate (Corning 50–013-PB) or DMEM with 1 mM pyruvate (Sigma, P8574) as indicated in the figure legends where “DMEM” refers to DMEM with 1 mM pyruvate, supplemented with 3.7 g/L sodium bicarbonate, 10% dialyzed FBS (Sigma-Aldrich, F0392) and 1% penicillin–streptomycin solution. To start an experiment, cells were trypsinized (ThermoFisher, 25200056), resuspended in media, counted using a coulter counter (Beckman Coulter, Multisizer 4) and seeded onto 24-well dishes (Nunc, 142475) with an initial seeding density of 15,000, 18,000, 50,000, or 20,000 cells/well for H1299, 143B, HCT116, H1299/143B GOT1/2 DKO, respectively. After 24 hours of incubation, the plates were moved into an Incucyte S3 (Sartorius) live cell imaging platform inside a humidified incubator at 37°C with 5% CO_2_ for a pre-treatment scan. Once the scan was complete, plates were removed for treatment. Drug treatments such as Atpenin A5 (MedChemExpress, HY-126653) and Rotenone (Sigma-Aldrich, R8875) were spiked-in as 2x solutions in DMEM without pyruvate and dialyzed FBS along with 2x sodium pyruvate. For treatments with varying media aspartate (Sigma-Aldrich, A7219) wells were washed with PBS, and filled with DMEM containing the various aspartate concentrations. For plates receiving asparagine (Sigma-Aldrich, A7094) or uridine (Sigma-Aldrich, U3003), this stocks were generated in water and made into 2x stocks in media, before a final dilution into experimental media so that the final concentration was 500 μM (Asn) or 200 μM (Uri), with vehicle wells receiving an equivalent volume of media with water in the place of the metabolite additives. Adenine (Sigma, A2786) was prepared fresh for each experiment by dissolving powder in 500 μL 1M HCl, neutralizing with 500 μL 1M NaOH, and filtering through a 0.22 μm filter (Fisher, FB12566506). This solution was then added to fresh media so that the final concentration was 100 μM. Live cell imaging was performed on the Incucyte S3 using the GFP and RFP channels with default exposure times. Images were processed using the associated Incucyte software to subtract background, define areas of cell confluence and GFP/RFP signal and extract the sum of the fluorescence intensity in these areas. The jAspSnFR3 signal (GFP channel) was normalized to an RFP signal as a stably expressed nuclear localized RFP (NucRFP). RFP counts were normalized to the pre-treatment scan and used to estimate cell counts per well by counting the number of nuclear foci in each field of view when scanning in the RFP channel. The normalized values for GFP/RFP and RFP count were exported and used to plot temporal aspartate and nuclei plots. NucRFP counts were also used to calculate proliferation rate in doublings per day with the following equation: Proliferation rate (doublings per day, 1/d) = (log_2_(final cell count / initial cell count))/days.

### Conventional Proliferation Assays and Cell Volume Measurements

Cells were trypsinized, resuspended in media, and counted (Beckman Coulter Counter Multisizer 4 or Nexcelom Auto T4 Cell-o-meter) and seeded overnight onto 24-well dishes (Corning, 3516) with the same initial seeding densities described above. After overnight incubation, 3 wells were counted for a starting cell count at the time of treatment. Cells were washed in phosphate-buffered saline (PBS) and 2 mL of treatment media was added to each well. Experiments were conducted in DMEM without pyruvate supplemented with 3.7 g/L sodium bicarbonate 10% dialyzed FBS and 1% penicillin-streptomycin solution, with or without 1 mM sodium pyruvate, 20 mM aspartate, or 0.5 mM Asparagine, 200 μM uridine, 100 μM adenine, or 10–25 mM succinic acid (Sigma, S3674). Drug treatments included rotenone (Sigma-Aldrich, R8875), cycloheximide (Sigma, C7698), Atpenin A5 (MedChemExpress, HY-126653), BAY-1895344 (Selleckchem, S8666) and DMSO as a vehicle (D2650). Cells were incubated in a humidified incubator at 37°C with 5% CO_2_ then counted after 3–4 days. Proliferation rate was determined as described above.

### Generation of KO cells

Knockout cell lines were generated as previously described^2,12^. Three single guide RNA (sgRNA) sequences targeting fumarate hydratase (FH) were purchased (Synthego) and are listed in the table below. Each sgRNA was resuspended in nuclease-free water, combined with SF buffer (Lonza, V4XC-2032), and sNLS-spCas9 (Aldevron, 9212). 2×10^5^ 143B cells were resuspended in the resulting solution containing ribonucleoprotein complexes (RNPs) and electroporated using a 4D-Nucleofector (Amaxa, Lonza) program FP-133. Nucleofected cells were then moved to a 12-well plate (Corning, 3513) and, after achieving confluence, were single-cell cloned by limiting dilution by plating 0.5 cells/well in a 96 well plate. Gene knockout was confirmed using western blots on the nucleofected pool and each single cell clone used in this study. GOT1/2 DKO cells were generated in the same way and previously described^2^.

**Table:** 

Guide	Sequence 5’ → 3’
FH sgRNA1	AGGCAAGCCAAAAUUCCUUC
FH sgRNA2	GGUACAUAUUCUAUCCGGA
FH sgRNA3	CAAAGGUAUCAUAUUCUAUC

### Western Blotting

Protein lysates were harvested in RIPA buffer (Sigma, R0278) supplemented with protease inhibitors (Fisher, A32953) and phosphatase inhibitors (Fisher, 78442). Protein concentration was determined using a Bicinchoninic Acid Assay (Fisher, 23225) using bovine serum albumin (BSA) as a protein standard. Equal amounts of protein were denatured with Bolt 4x Loading Dye (ThermoFisher, B0007) and Bolt 10x reducing agent (ThermoFisher, B0004), heated at 95°C for 5 min, and loaded onto 4–12% by SDS-polyacrylamide gels (Invitrogen, NW04127). After electrophoretic separation, proteins were transferred onto a 0.22 mm nitrocellulose using iBlot2 transfer stacks (Fisher, IB23001) and transferred with the P0 system setting. Membranes were blocked with 5% BSA in Tris-buffered saline with 0.1% Tween-20 (TBS-T) and incubated at 4°C overnight with the following antibodies: anti-GOT2 (Proteintech, 14800–1-AP, 1:1000), anti-GOT1 (Cell Signaling, 34423S, 1:1,000), anti-GFP (Sigma, 1:1000), anti-FH (Origene, TA500675S, 1:1000), anti-SDHB (Atlas, HPA002868, 1:1,000), anti-pChk1 (Cell Signaling, 2348S, 1:1000), anti-pChk2 (Cell Signaling, 2197S, 1:1000), anti-GAPDH (Cell Signaling, 5174S, 1:5000), and anti-Vinculin (Sigma, SAB4200729, 1:10,000). The next morning, membranes were washed three times with TBS-T and the following secondary antibodies were added: 800CW Goat anti-Mouse IgG (LiCOR, 926–32210; 1:15,000), 680RD Goat anti-Rabbit IgG (LiCOR, 926–68071; 1:15,000). Membranes were washed three more times with TBS-T and imaged on a LiCOR Odyssey Near-Infrared imaging system.

### RNA Extractions and tRNA Aminoacylation Quantification

143B and H1299 cells were grown on 6 well plates in DMEM without pyruvate, in dialyzed FBS. For the Atpenin A5 treatment, 1 mM pyruvate was added to the media. At 50% confluency, cells were treated with drugs in replicates for 30 hours. H1299 cells were treated with vehicle (DMSO), rotenone (100 nM), or Atpenin A5 (5 μM). 143B cells were treated with vehicle (DMSO), rotenone (50 nM), or Atpenin A5 (5 μM). For RNA extraction, the media on the cells was quickly and thoroughly aspirated before adding 3 mL TRIzol (ThermoFisher, A33250) to cover all the cells. From this point onward, everything was kept ice cold to prevent hydrolysis of the aminoacylation. After a 2 min incubation, the cell material was scraped down the slope mixing it with the TRIzol, then 2×1.5 mL was transferred to 2 mL Eppendorf tubes and 0.3 mL chloroform was added. The tubes were vortexed 2 min and then centrifuged (17,000×*g*, 5 min). From each tube, 0.75 mL of the upper layer was transferred to a tube with 0.8 mL isopropanol (IPA) (ThermoFisher, A464SK), then mixed and incubated 60 min at −20°C. Tubes were then centrifuged (17,000×*g*, 15 min) and RNA pellets were washed twice with 1 mL 80% IPA containing 100 mM sodium acetate (pH = 4.5) (Sigma, S7545). Washes are critical to prevent glycerol present in TRIzol from inhibiting the subsequent periodate oxidation step. A last wash was performed using 1 mL 100% IPA and after removing the supernatant the RNA pellets were air-dried at room temperature, then stored dry at −80°C. Aminoacylation levels, referred to as “charge”, were measured by sequencing, determining the fraction of tRNAs protected from periodate oxidation and 3’ nucleotide cleavage, as previously described in detail^75^. The values for each of the multiple tRNA transcripts decoding each codon were calculated as an expression weighted average of all codon-specific transcript charges.

### Media Uptake Flux

The cells were maintained for at least four passages with a 1/20 split at each passage in DMEM with dialyzed FBS and the tracer and/or metabolites used during the uptake experiment. For GOT1/2 DKO 143B cells expressing SLC1A3, 500 μM aspartate was added. For 143B WT cells, 100 μM U-^13^C Asn was added to achieve a steady-state label fraction in the proteome. To start the experiment, cells were seeded on 6 well dishes at 100,000 cells/well. On the next day fresh media was added and t=0 media samples were collected. Cells were then incubated, and subsequent media samples collected at the indicated time. After the last media collection, the residual media volume was quantified to correct for evaporation. For U-^13^C Asn tracing, the labelling ratio was determined by extracting intracellular metabolites after the last media collection. Two dishes were run in parallel and used for counting to determine proliferation rates and cell volume measurement using a Coulter Counter.

### Asparagine Flux Calculations

143B cells do not have appreciable asparagine deaminase activity, so it can be assumed that, in the presence of media containing ^13^C-asparagine, intracellular ^13^C-asparagine derives from influx while ^12^C asparagine derives from synthesis. Using media sampling to ascertain net influx of ^13^C Asn (J_in_) and efflux of ^12^C Asn (J_out_) and the intracellular ratio of labeled to unlabeled Asn (^13^C-Asn/Asn) in the cell, the remaining flux into protein synthesis (the only net consumption fate for asparagine) can be resolved. Assuming stable intracellular asparagine pools then: (J_in_ + J_syn_ = J_prot_ + J_out_), meaning (J_prot_ = J_in_ + J_syn_ - J_out_). Since intracellular (^13^C-Asn/Asn) = J_in_/(J_syn_-J_out_), these equations can be combined and rearranged to J_prot_ = J_in_(1+(Asn/^13^C-Asn)).

### Acid Hydrolysis to Measure Amino Acids and Pyrimidines

Two 12 well plates were seeded in parallel in DMEM without pyruvate, with dialyzed FBS. At 90% confluency, plates were washed thrice with saline (Fisher, 23293184), then to one plate 1 mL 6 M HCl (Sigma, 84429) was added to each well. The plate was sealed with adhesive PCR plate seal (ThermoFisher, AB0558) and placed in an incubator at 90°C for 20 hours for hydrolysis. Meanwhile, each well on the other plate was trypsinized and counted on a Coulter counter. At the end of the 20-hour incubation, the hydrolysate was moved to a tube followed by thrice washing with 1 mL HPLC grade water to collect all material. This was dried down and reconstituted again in 0.5 mL 6 M HCl following another incubation at 90°C for 48 hours to complete the hydrolysis. Tubes were dried and reconstituted again in 0.5 mL 6 M HCl, then a volume equivalent to 20,000 cells was moved to a fresh tube and dried down. To removed water insoluble material, tubes were reconstituted with 1 mL HPLC grade water and centrifuged (17,000g, 15 mins) before moving 0.5 mL to a fresh tube and drying. Dried samples were reconstituted with 40 μL 80% HPLC grade methanol, containing internal standards for both amino acids and nucleotides/nucleobases (Cambridge Isotope Laboratories, MSK CAA-1; Cambridge Isotope Laboratories, CLM-8400-PK).

### Absolute Quantification by Isotope Dilution

Dried samples were reconstituted with 40 μL 80% HPLC grade methanol containing 5 μM U-^13^C, U-^15^N labelled canonical amino acid mix (Cambridge Isotope Laboratories, MSK CAA-1) and transferred to vials for measurement by LCMS. For pyrimidine nucleobase/nucleoside quantification a U-^13^C internal standard was made by partial hydrolysis (12 hours in 6 M HCl at 90°C) of U-^13^C spirulina whole cells lyophilized powder (Cambridge Isotope Laboratories, CLM-8400-PK). The peak area for each compound was divided by its labelled standard to derive the response ratio. The response ratio was then mapped to a calibration curve to infer the compound concentration in the vial. The intracellular concentration of each samples was calculated by finding the molar amount using the concentration in the vial multiplied by the dilution and dividing by the total cell volume. To make the calibration curves a non-labelled amino acid mixture was made from an analytical amino acid standard without glutamine and asparagine (Sigma, A9906) and added glutamine (Sigma, 76523) and asparagine (Sigma, 51363) to match the concentration of the other amino acids. For pyrimidine nucleobase/nucleoside quantification this pool was also mixed with equimolar uracil (Sigma, U1128), uridine (Sigma, U3003), 2-deoxyuridine (Sigma, D5412), thymine (Sigma, T0376), cytosine (Sigma, C3506) and cytidine (Cayman Chemical, 29602). Using this mix, three replicates of a 12 point 2-fold dilution series was made with a maximum concentration of 500 μM and a volume per dilution of 40 μL. These were placed in a centrivap until dry and reconstituted with 40 μL 80% HPLC grade methanol containing the appropriate isotopic internal standard and transferred to vials for measurement by LCMS. The peak area for each compound was divided by its labelled standard to derive the response ratio, then the best fitting calibration curves for each compound were chosen among either linear, power or a second-degree polynomial. Each calibration curve was manually inspected for proper fit and measurements below or above the concentration range of the dilution series were discarded.

### Aspartate Fate Flux Calculations

Total cell concentrations for Asp+Asn and pyrimidine metabolites were converted into fluxes using the equation F_i_ = K ln(2)C_i_ where Fi is the flux (mM/h) for compound *i*, K is the cell-volume proliferation rate (1/h), and C_i_ is the total cell concentration for compound *i* (mM). This equation assumes that cells are undergoing constant exponential proliferation with an associated constant rate of biomass accumulation and constant cell volume. From this, aspartate flux towards protein was determined by subtracting the contribution from asparagine to the Asp+Asn pool. Potential aspartate consumption towards arginine was determined to be absent, as shown in Extended Data Fig. 4c. The total cell concentration of purines could not be determined due to their sensitivity to hydrolysis (data not shown). Therefore, purine abundance was estimated to be equal to pyrimidine abundance, consistent with published measurements^29,30^. *De novo* purine synthesis consumes aspartate once while generating the shared precursor IMP and once more for conversion of IMP to AMP. It was assumed that AMP and GMP synthesis flux is equal, and thus the extra aspartate consumed for half of the purine demand yielded a total aspartate consumption in *de novo* purine synthesis that is 1.5 times that of pyrimidine synthesis.

### Polar Metabolite Extractions

Time-course. For LC-MS measurements across several time-points, 143B cells were seeded overnight at either 2×10^5^, 1×10^5^, or 0.5×10^5^ cells per well of a 6-well dish for the 10/16hr, 24hr, and 44hr time points, respectively. The next day, cells were washed twice with PBS and changed to the indicated medias supplemented with 1 mM pyruvate, 10% dialyzed FBS, 1% penicillin-streptomycin, and treatments as indicated, and returned to the tissue culture incubator. After 6 hours, polar metabolites were extracted from cells by three washes with ice-cold blood bank saline, (Fisher, 23293184) then 300 μL of 80% HPLC grade methanol in HPLC grade water was added to each well and cells were scraped with the back of a P1000 pipet tip and transferred to Eppendorf tubes. Tubes were centrifuged (17,000xg, 15 mins, 4°C) and the supernatant containing polar metabolites was transferred to a new centrifuge tube and placed in a centrivap until lyophilized. Corresponding plates with the same treatment conditions were trypsinized at the same time metabolites were extracted and counted on a Beckman Coulter Counter to obtain total cell volume per well. Average cell volumes for each condition were calculated and used to normalize metabolites after centrifugation. Dried metabolites were resuspended in 40 μL solvent per μL cell volume in 80% HPLC grade MeOH containing ^13^C-labeled metabolites made by partial hydrolysis (12 hours in 6 M HCl at 90°C) of U-^13^C spirulina whole cells lyophilized powder (Cambridge Isotope Laboratories, CLM-8400-PK) as an internal standard to account for matrix effects and absolute quantitation of intracellular metabolites. Ion counts were normalized to the internal standard metabolite when appropriate to determine a response ratio. *Succinate and Aspartate Concentration Measurements*. A standard curve of known succinate and aspartate concentrations over three orders of magnitude was generated in 80% HPLC-grade MeOH containing ^13^C spirulina standard and run in parallel with the time-course experiment detailed above. Ion counts for succinate were normalized by cell volume and intracellular concentrations were calculated according to the standard curve. *Other Measurements*. For standard metabolic analysis, cells were seeded overnight at 1×10^5^ cells per well of a 6-well dish. The next day, cells were washed twice with PBS and changed to the indicated medias supplemented with 10% dialyzed FBS, 1% penicillin-streptomycin, and treatments as indicated, and returned to the tissue culture incubator. After 30–32 hours, polar metabolites were extracted from cells by the same protocol as mentioned above.

### Media Metabolite Extraction

For media metabolite extraction, 10 μL media was sampled, added to 990 μL 80% HPLC grade methanol in HPLC grade water and incubated at −20°C for 30 min. Tubes were centrifuged (17,000g, 15 mins, 4°C), 400 μL of the supernatant containing media metabolites was transferred to a new tube and placed in a centrivap until dry. Dried samples were reconstituted with 40 μL 80% HPLC grade methanol, containing internal standards when appropriate, and transferred to vials for measurement by LCMS.

### Isotope Tracing

^*15*^*N Glutamine tracing*. The fractional contribution of individual components into their respective aspartate consuming fate was determined in 143B and H1299 cells for the salvageable metabolites asparagine (Asn), uridine (Uri), adenine (Ade), hypoxanthine (Hpx) (Cayman, 22254), and guanine (Gua) (Sigma, G11950), along with a vehicle treatment (Vec). The salvageable metabolites were spiked in from a 20x stock solution to achieve a final concentration of: 500 μM Asn, 200 μM Uri, 100 μM Hpx, 100 μM Ade or 100 μM Gua. The fraction of salvage was determined by stable isotope tracing, performed using both Gln amide-^15^N (Cambridge Isotope Laboratories, NLM-557-PK) and Gln alpha-^15^N (Cambridge Isotope Laboratories, NLM-1016-PK) in separate reactions and added to DMEM without glucose, glutamine, pyruvate and phenol red (Sigma, D5030) supplemented with 10% dialyzed FBS, 1x penicillin-streptomycin and 25 mM glucose (Sigma, G7528). The combination of cell lines, salvageable metabolites and tracers gave 24 conditions which were labelled to steady state by culturing for four passages with a 1/20 split at each passage. At the end of the last passage each condition was split into four technical replicates and plated on 24 well plates. Upon reaching confluency, polar metabolites were extracted and submitted to LC-MS. The relative contribution of guanine salvage into the GTP pool was determined using the m+0 vs. m+3 GTP isotopologue fractions from the Gln amide-^15^N labelled samples. The relative contribution of both adenine and hypoxanthine salvage into the ATP pool was determined using the m+0 ATP isotopologue fraction from the Gln amide-^15^N labelled samples and the direct contribution from adenine was determined using the m+1 isotopologue fractions of aspartate compared to ATP from the Gln alpha-^15^N labelled samples. *U-*^*13*^*C Glutamine tracing*. WT 143B cells were seeded at 1×10^5^ cells per well of a 6-well dish. The next morning, cells were washed twice with PBS and changed to DMEM without glucose, glutamine, pyruvate, or phenol red (Sigma, D5030) supplemented with 10% dialyzed FBS, 1% penicillin-streptomycin, 1 mM pyruvate, 25 mM ^12^C glucose (Sigma, G7528), and 4 mM U-^13^C glutamine (Cambridge Isotopes, CLM-1822). 143B cells were treated as indicated for 32 hours, then harvested by the same protocol detailed above.

### Liquid Chromatography-Mass Spectrometry (LC-MS)

Resuspended samples were transferred to liquid chromatography-mass spectrometry (LCMS) vials for measurement by LCMS. Metabolite quantitation was performed using a Q Exactive HF-X Hybrid Quadrupole-Orbitrap Mass Spectrometer equipped with an Ion Max API source and H-ESI II probe, coupled to a Vanquish Flex Binary UHPLC system (Thermo Scientific). Mass calibrations were completed at a minimum of every 5 days in both the positive and negative polarity modes using LTQ Velos ESI Calibration Solution (Pierce). Polar Samples were chromatographically separated by injecting a sample volume of 1 μL into a SeQuant ZIC-pHILIC Polymeric column (2.1 × 150 mm 5 mM, EMD Millipore). The flow rate was set to 150 mL/min, autosampler temperature set to 10 °C, and column temperature set to 30 °C. Mobile Phase A consisted of 20 mM ammonium carbonate and 0.1 % (v/v) ammonium hydroxide, and Mobile Phase B consisted of 100 % acetonitrile. The sample was gradient eluted (%B) from the column as follows: 0–20 min.: linear gradient from 85 % to 20 % B; 20–24 min.: hold at 20 % B; 24–24.5 min.: linear gradient from 20 % to 85 % B; 24.5 min.-end: hold at 85 % B until equilibrated with ten column volumes. Mobile Phase was directed into the ion source with the following parameters: sheath gas = 45, auxiliary gas = 15, sweep gas = 2, spray voltage = 2.9 kV in the negative mode or 3.5 kV in the positive mode, capillary temperature = 300 °C, RF level = 40 %, auxiliary gas heater temperature = 325 °C. Mass detection was conducted with a resolution of 240,000 in full scan mode, with an AGC target of 3,000,000 and maximum injection time of 250 msec. Metabolites were detected over a mass range of 70–1050 *m/z*. Quantitation of all metabolites was performed using Tracefinder 4.1 (Thermo Scientific) referencing an in-house metabolite standards library using ≤ 5 ppm mass error. Data from stable isotope labeling experiments includes correction for natural isotope abundance using IcoCor software v.2.2.

### Data Analysis

All graphs and statistical analyses were made in GraphPad Prism 10.4.1. Technical replicates, defined as parallel biological samples independently treated, collected, and analyzed during the same experiment, are shown. Experiments were verified with ≥ 2 independent repetitions showing qualitatively similar results. Details pertaining to all statistical tests can be found in the figure legends.

## Supplementary Material

Supplement 1 **Extended Data Fig. 1. Measurements of aspartate levels and cell proliferation in contexts where aspartate acquisition is constrained.** a. GFP/RFP ratio of H1299 jAspSnFR3/NucRFP cells treated with a rotenone titration in DMEM without pyruvate (n=4).
b. NucRFP counts per well of H1299 jAspSnFR3/NucRFP cells treated with a rotenone titration in DMEM without pyruvate (n=4).
c. Aspartate levels in H1299 cells measured by LC-MS 2–5 days post treatment with a rotenone titration and rescued by cotreatment of 1mM pyruvate in DMEM (n=2). Levels were normalized to values from untreated cells extracted on day 0.
d. Cell counts of H1299 cells measured 2–5 days post treatment with a rotenone titration cultured in DMEM without pyruvate, with one condition cotreated with 1 mM pyruvate, normalized to start count (n=2).
e. Total rotenone ion counts measured by LC-MS 2–5 days post treatment in H1299 cells treated with a rotenone titration in DMEM without pyruvate, with one condition cotreated with 1 mM pyruvate in DMEM (n=2).
f. Western blot verifying GOT1/2 double knockout (DKO) in 143B and H1299 cells. GFP antibody also shows that 143B and H1299 cells with jAspSnFR3 (jAsp)/NucRFP express jAspSnFR3 (epitope shared with GFP). Vinculin is used as a loading control.
g. Proliferation rates of GOT1/2 DKO 143B cells treated with a titration of environmental aspartate in DMEM without pyruvate (n=3).
h. Proliferation rates of GOT1/2 DKO H1299 cells treated with a titration of environmental aspartate for 96 hours in DMEM without pyruvate (n=3).
i. GFP/RFP ratio of GOT1/2 DKO H1299 jAspSnFR3/NucRFP cells against a titration of environmental aspartate concentrations for 96 hours in DMEM without pyruvate (n=4).
j. NucRFP counts of GOT1/2 DKO H1299 jAspSnFR3/NucRFP cells against a titration of environmental aspartate concentrations in DMEM without pyruvate (n=4).
k. GFP/RFP ratio of 143B jAspSnFR3/NucRFP cells treated with Vehicle (DMSO) or 1 μg/mL cycloheximide (CHX) either at the start of the assay (0 hrs) or 24 hrs after initial treatment in DMEM without pyruvate.
l. NucRFP counts per well of 143B jAspSnFR3/NucRFP cells treated with Vehicle (DMSO) or 1 μg/mL cycloheximide (CHX) either at the start of the assay (0 hrs) or 24 hrs after initial treatment in DMEM without pyruvate.
m. GFP/RFP ratio of 143B jAspSnFR3/NucRFP cells treated with an AA5 titration in DMEM without pyruvate (n=4).
n. NucRFP counts per well of 143B jAspSnFR3/NucRFP cells treated with an AA5 titration in DMEM without pyruvate (n=4).
o. GFP/RFP ratio of 143B jAspSnFR3/NucRFP cells treated with either Veh or 5 μM AA5 −/+ 20 mM aspartate in DMEM (n=3).
Data are plotted as means ± standard deviation (SD) (a-b) or means ± standard error of the mean (SEM) (i-o).
**Extended Data Fig. 2. Aminoacylation charge measurements of aspartate-related tRNAs upon treatments to deplete aspartate.** a. Percentage of aminoacylation (tRNA) charge of cytosolic and mitochondrial aspartate and asparagine in 143B cells treated with Vehicle (Veh), 50 nM Rotenone or 5 μM AA5 for 30 hours (n=2). For AA5 treatment, 1 mM pyruvate was supplemented to the DMEM without pyruvate base media. X axis labels: mito refers to mitochondrial tRNA sequences while its absence indicates cytosolic tRNA sequences, Asp/Asn refers to the cognate amino acid, terminal three letter codes refer to the codons for the cognate amino acid.
b. Percentage of aminoacylation (tRNA) charge of cytosolic and mitochondrial aspartate and asparagine in H1299 cells subjected to Veh, 100 nM Rotenone or 5 μM AA5 after 30 hours (n=2). For AA5 treatment, 1 mM pyruvate was supplemented to the DMEM without pyruvate base media.
c. Percentage of aminoacylation (tRNA) charge of cytosolic and mitochondrial aspartate and asparagine in GOT1/2 DKO 143B cells subjected to a titration of environmental aspartate concentrations (n=2).
d. Percentage of aminoacylation (tRNA) charge of cytosolic and mitochondrial aspartate and asparagine in GOT1/2 DKO H1299 cells subjected to a titration of environmental aspartate concentrations (n=2).
Data are plotted as means ± standard deviation (SD).
**Extended Data Fig. 3. Metabolite levels for pyrimidine synthesis pathway intermediates upon SDH inhibition over time.** a. GMP levels measured by LC-MS of 143B cells treated with either Veh (DMSO) or 5 μM AA5 in DMEM extracted at 24 hours and 44 hours post treatment, normalized to Veh (n=3).
b. Dihydroorotate levels measured by LC-MS of 143B cells treated with either Veh (DMSO) or 5 μM AA5 in DMEM extracted at 24 hours and 44 hours post treatment, normalized to Veh (n=3).
c. UMP levels measured by LC-MS of 143B cells treated with either Veh (DMSO) or 5 μM AA5 in DMEM extracted at 24 hours and 44 hours post treatment, normalized to Veh (n=3).
d. UDP levels measured by LC-MS of 143B cells treated with either Veh (DMSO) or 5 μM AA5 in DMEM extracted at 24 hours and 44 hours post treatment, normalized to Veh (n=3).
Data are plotted as means ± standard deviation (SD). Statistics in this figure were generated using a two-way ANOVA with multiple comparisons and p-values for highlighted comparisons are shown above the horizontal lines on each plot.
**Extended Data Fig. 4. Identification of aspartate-fate supplementation conditions that obviate the need for aspartate consumption for their acquisition.** a. Top diagram shows glutamine (Gln) amide-^15^N label incorporation into asparagine (Asn) and the bottom graph shows the Gln amide-^15^N label incorporation into Asn at steady-state for cell lines 143B and H1299 grown in DMEM supplemented with vehicle (Vec), or 500 μM Asn (n=4, plotted as grouped bars).
b. Top diagram shows Gln amide-^15^N label incorporation into pyrimidines and the bottom graph shows the Gln amide-^15^N label incorporation into UTP at steady-state for cell lines 143B and H1299 grown in DMEM supplemented with vehicle (Vec), or 500 μM uridine (Urd) (n=4, plotted as grouped bars).
c. Top diagram shows Gln alpha-^15^N label incorporation into aspartate (Asp) and subsequently arginine (Arg). Bottom isotopologue distributions for Gln, Asp, and Arg at steady state for cell lines 143B and H1299 grown in standard DMEM without pyruvate (n=4, plotted as grouped bars).
d. Top diagram shows Gln amide-^15^N label incorporation into purines and the resulting changes following salvage of hypoxanthine (Hpx), adenine (Ade) or guanine (Gua). ATP and GTP are either derived from *de novo* synthesis or salvage. ATP can be salvaged from adenine directly or through hypoxanthine/IMP as a product of adenine/AMP deamination. Bottom isotopologue distributions show Gln amide-^15^N label incorporation into GTP and ATP at steady-state for cell lines 143B and H1299 grown in DMEM supplemented with vehicle (Vec) or 100 μM Hpx, Ade or Gua (n=4, plotted as grouped bars).
**Extended Data Fig. 5. Uridine supplementation impacts aspartate homeostasis in SDH impaired conditions.** a. NucRFP counts per well of 143B jAspSnFR3/NucRFP cells treated with either Veh (DMSO), 5 μM AA5, AA5 +500 μM Asn, AA5 +100 μM adenine (Ade), or AA5 +200 μM uridine (Uri) in DMEM (n=4).
b. Heatmap of the proliferation rates (in doublings per day per six-hour window) of 143B jAspSnFR3/NucRFP cells treated with either Veh (DMSO), 5 μM AA5, AA5 +500 μM Asn, AA5 +100 μM adenine (Ade), or AA5 +200 μM uridine (Uri) in DMEM (n=4).
c. GFP/RFP of 143B jAspSnFR3/NucRFP cells treated with either Veh (DMSO), 50 nM Rotenone, or 50 nM Rotenone +200 μM Uri in DMEM without pyruvate (n=4).
d. NucRFP counts per well of 143B jAspSnFR3/NucRFP cells treated with either Veh (DMSO), 50 nM Rotenone, or 50 nM Rotenone +200 μM Uri in DMEM without pyruvate (n=4).
e. GFP/RFP of H1299 jAspSnFR3/NucRFP cells treated with either Veh (DMSO), 1.5 μM AA5, or 1.5 μM AA5 +200 μM uridine (Uri) in DMEM (n=4).
f. NucRFP counts per well of H1299 jAspSnFR3/NucRFP cells treated with either Veh (DMSO), 1.5 μM AA5, or 1.5 μM AA5 +200 μM uridine (Uri) in DMEM (n=4).
g. GFP/RFP of HCT116 jAspSnFR3/NucRFP cells treated with either Veh (DMSO), 5 μM AA5, or 5 μM AA5 +200 μM uridine (Uri) in DMEM (n=4).
h. NucRFP counts per well of HCT116 jAspSnFR3/NucRFP cells treated with either Veh (DMSO), 5 μM AA5, or 5 μM AA5 +200 μM uridine (Uri) in DMEM (n=4).
Data are plotted as means ± standard deviation (SD) (a, e-h) or means ± standard error of the mean (SEM) (c-d).
**Extended Data Fig. 6. Quantification of aspartate metabolic demands.** a. Influx/efflux of amino acids from DMEM without pyruvate in GOT1/2 DKO 143B cells expressing Glu/Asp transporter SLC1A3. Media was spiked with 500 μM aspartate (Asp) to measure its uptake. Cells and media were sampled at 23h and 31h (hours) (n=3).
b. Influx/efflux of amino acids from DMEM without pyruvate in WT 143B cells. Media was spiked with U-^13^C Asn (*Asn in the plot, Asn without asterisk is unlabeled Asn flux) to measure asparagine uptake in 143B cells at 10h and 24h (n=3).
c. Diagram of asparagine (Asn) fluxes in a cell. Net influx of U-^13^C labelled Asn (*J*_in_), net efflux of unlabeled Asn (*J*_out_), net deposition of Asn into protein (*J*_prot_) and Asn synthesis from Asp (*J*_syn_). Unlabeled metabolites are symbolized by green and U-^13^C labelling is symbolized by blue.
d. Measured Asn consumption fluxes in 143B cells at 10h and 24h using the media uptake data from (b). Asn is consumed into protein synthesis (Protein) and leaked into the media (Efflux). Efflux is calculated as the initial rate, assuming no Asn is provided in the media (n=3).
e. Total amino acids and pyrimidines liberated by acid hydrolysis of 143B and H1299 cells, normalized by total cell volume. Gln and Asn are converted during acid hydrolysis to glutamate (Glu) and Asp, respectively. Glycine is not shown because it is also produced from purine hydrolysis. Abbreviations: Cyt, cytidine; Ura, uracil; Thy, thymidine.
f. Relative consumption flux towards each fate of aspartate in 143B cells, estimated using best estimates from (d) and (e). Synthesis of purines was assumed equal to pyrimidines and thus aspartate consumption towards purines was estimated 1.5 times the consumption towards pyrimidines to account for two aspartate molecules consumed for AMP and one for GMP (see Extended Data Fig. 4d). *Asn efflux is calculated as the initial rate for cells are grown in DMEM which does not contain Asn. Blue bars indicate that the aspartate fate is a nitrogen donation, yielding fumarate while red bars indicate that the fate consumes aspartate carbons.
Data are plotted as means ± standard deviation (SD).
**Extended Data Fig. 7. Complex I inhibition decreases aspartate levels over time while restoring pyrimidine synthesis in SDH impaired cells.** a. Aspartate levels measured by LC-MS in 143B cells treated with either Veh (DMSO), 5 μM AA5, or 5 μM AA5 +50 nM Rotenone in DMEM 24, 44, and 66 hours post treatment (n=3).
b. Relative ion counts for all aspartate isotopologues derived from U-^13^C glutamine measured by LC-MS treated with either Veh (DMSO), 5 μM AA5, or 5 μM AA5 +50 nM Rotenone in DMEM for 32 hours (n=3).
c. Relative ion counts for all succinate isotopologues derived from U-^13^C glutamine measured by LC-MS treated with either Veh (DMSO), 5 μM AA5, or 5 μM AA5 +50 nM Rotenone in DMEM for 32 hours (n=3).
d. Relative ion counts for all carb-Asp isotopologues derived from U-^13^C glutamine measured by LC-MS treated with either Veh (DMSO), 5 μM AA5, or 5 μM AA5 +50 nM Rotenone in DMEM for 32 hours (n=3).
e. Relative ion counts for all dihydroorotate (DHO) isotopologues derived from U-^13^C glutamine measured by LC-MS treated with either Veh (DMSO), 5 μM AA5, or 5 μM AA5 +50 nM Rotenone in DMEM for 32 hours (n=3).
f. Relative ion counts for all orotate (Oro) isotopologues derived from U-^13^C glutamine measured by LC-MS treated with either Veh (DMSO), 5 μM AA5, or 5 μM AA5 +50 nM Rotenone in DMEM for 32 hours (n=3).
g. Relative ion counts for M+0–4 UMP isotopologues derived from U-^13^C glutamine measured by LC-MS treated with either Veh (DMSO), 5 μM AA5, or 5 μM AA5 +50 nM Rotenone in DMEM for 32 hours (n=3).
Data are plotted as means ± standard deviation (SD).
**Extended Data Fig. 8. SDH inhibition in Fumarate Hydratase deficient cells impairs aspartate utilization into pyrimidine synthesis.** a. Western blot validating complete loss of fumarate hydratase (FH) in two 143B single cell clones while SDH is still intact. GAPDH is used as a loading control.
b. Fumarate abundance measured by LC-MS in FH KO 143B cells treated with either DMSO (Veh) or 5 μM AA5 in DMEM after 48 hours, normalized to vehicle (n=3).
c. Succinate abundance measured by LC-MS in FH KO 143B cells treated with either DMSO or 5 μM AA5 in DMEM after 48 hours, normalized to vehicle (n=3).
d. Aspartate abundance measured by LC-MS in FH KO 143B cells treated with either DMSO or 5 μM AA5 in DMEM after 48 hours, normalized to vehicle (n=3).
e. Carb-Asp abundance measured by LC-MS in FH KO 143B cells treated with either DMSO or 5 μM AA5 in DMEM after 48 hours, normalized to vehicle (n=3).
f. UTP abundance measured by LC-MS in FH KO 143B cells treated with either DMSO or 5 μM AA5 in DMEM after 48 hours, normalized to vehicle (n=3).
g. CTP abundance measured by LC-MS in FH KO 143B cells treated with either DMSO or 5 μM AA5 in DMEM after 48 hours, normalized to vehicle (n=3).
h. Asparagine abundance measured by LC-MS in FH KO 143B cells treated with either DMSO or 5 μM AA5 in DMEM after 48 hours, normalized to vehicle (n=3).
i. AMP abundance measured by LC-MS in FH KO 143B cells treated with either DMSO or 5 μM AA5 in DMEM after 48 hours, normalized to vehicle (n=3).
Data are plotted as means ± standard deviation (SD) and compared with a paired two-tailed student’s t-test. P-values for the highlighted comparisons are shown above the horizontal lines on each plot.
**Extended Data Fig. 9. SDH inhibition causes replication stress signaling and dependence through impaired pyrimidine synthesis in diverse contexts.** a. Proliferation rates of 143B cells treated for 96 hours with an AA5 titration in DMEM (n=3).
b. Proliferation rates of H1299 cells treated for 72 hours with an AA5 titration in DMEM (n=3).
c. Proliferation rates of HCT116 cells treated for 72 hours with an AA5 titration in DMEM (n=3).
d. Median cell volumes of 143B cells treated for 72 hours with a rotenone titration in DMEM without pyruvate (n=3).
e. Median cell volumes of GOT1/2 DKO 143B cells treated for 72 hours with an aspartate titration in DMEM without pyruvate (n=3).
f. Median cells volumes of WT 143B cells and FH KO 143B cells after 72 hours in DMEM (n=3).
g. Median cell volumes of HCT116 cells treated for 72 hours with either DMSO, 1 μM AA5, 1 μM AA5 +20 mM aspartate, 1 μM AA5 +50 nM rotenone, or 1 μM AA5 +200 μM uridine in DMEM (n=3).
h. Median cell volumes of H1299 cells treated for 72 hours with either DMSO, 1.5 μM AA5, 1.5 μM AA5 +40 mM aspartate, 1.5 μM AA5 +50 nM rotenone, or 1.5 μM AA5 +200 μM uridine in DMEM (n=3).
i. Median cell volumes of FH KO 143B cells treated for 72 hours with either Veh (DMSO) 5 μM AA5, 5 μM AA5 +20 mM Asp, or 5 μM AA5 +200 μM uridine in DMEM (n=3).
j. Median cell volumes of 143B cells treated for 72 hours with either Veh, Veh with 10 or 25 mM succinate, 5 μM AA5, 5 μM AA5 + 50 nM rotenone, 5 μM AA5 + 50 nM rotenone with either 10 or 25 mM succinate, or 5 μM AA5 + 50 nM rotenone with 10 or 25 mM succinate and 200 μM uridine in DMEM (n=3).
k. Western blot showing phosphorylation of Chk1 and Chk2 in H1299 cells treated for the indicated time with either NT (DMSO), 1.5 μM AA5 (-), 1.5 μM AA5 +40 mM aspartate, 1.5 μM AA5 +50 nM rotenone, or 1.5 μM AA5 +200 μM uridine in DMEM. Vinculin is used as a loading control.
l. Western blot showing phosphorylation of Chk1 and Chk2 in 143B cells treated for the indicated time with either NT (DMSO), 5 μM AA5(-), 5 μM AA5 +20 nM BAY, or 5 μM AA5 +0.6 μM VE-821 in DMEM. Vinculin is used as a loading control.
m. Proliferation rates of 143B cells treated for 72 hours with either Veh (DMSO) or 5 μM AA5 with the indicated dose of BAY-1895344 in DMEM (n=3).
n. Proliferation rates of HCT116 cells treated for 72 hours with either Veh (DMSO) or 1 μM AA5 with or without 20 nM BAY-1895344 in DMEM (n=3).
o. Proliferation rates of H1299 cells treated for 72 hours with either Veh (DMSO) or 1.5 μM AA5 with or without 20 nM BAY-1895344 in DMEM (n=3).
p. Proliferation rates of 143B cells treated for 72 hours with either Veh (DMSO) or 50 nM rotenone with or without 20 nM BAY-1895344 in DMEM without pyruvate (n=3).
q. Relative proliferation rates of 143B cells after 72 hours of treatment: Veh (DMSO), 5 μM AA5, or 5 μM AA5 +200 μM uridine with the indicated dose of BAY-1895344 in DMEM (n=3). Each group is normalized to its respective vehicle control.
r. Proliferation rates of 143B cells after 72 hours of treatment with either Veh (DMSO), 5 μM AA5, 5 μM AA5 +20 mM aspartate, 5 μM AA5 +50 nM rotenone, or 5 μM AA5 +200 μM uridine with or without 1 μM VE-821 in DMEM (n=3).
Data are plotted as means ± standard deviation (SD). Statistics in this figure were generated either using an ordinary one-way ANOVA test (j) or an unpaired two tailed student’s t-test (n-p) with multiple comparisons and p-values for relevant comparisons are shown above the horizontal lines on each plot.

## Data Availability

All data supporting the findings of this study are available within the paper and its supplementary information. tRNA aminoacylation charge as shown in Extended Data Figure 2 is provided in the supplement for all 20 amino acids. For Extended Data Figure 6, relevant data, code, and analysis can be found at: https://github.com/krdav/lab-work/tree/main/H1299_143B_prot-nucl-quant/
