# Supplementary material for: Succinate Dehydrogenase loss causes cascading metabolic effects that impair pyrimidine biosynthesis
Source: bioRxiv. 2025 Feb 19:2025.02.18.638948. Preprint. [Version 1] doi: 10.1101/2025.02.18.638948 (PMC11870577; doi:10.1101/2025.02.18.638948)
Supplement: Supplement 1 — Extended Data Fig. 1. Measurements of aspartate levels and cell proliferation in contexts where aspartate acquisition is constrained. a. GFP/RFP ratio of H1299 jAspSnFR3/NucRFP cells treated with a rotenone titration in DMEM without pyruvate (n=4). b. NucRFP counts per well of H1299 jAspSnFR3/NucRFP cells treated with a rotenone titration in DMEM without pyruvate (n=4). c. Aspartate levels in H1299 cells measured by LC-MS 2–5 days post treatment with a rotenone titration and rescued by cotreatment of 1mM pyruvate in DMEM (n=2). Levels were normalized to values from untreated cells extracted on day 0. d. Cell counts of H1299 cells measured 2–5 days post treatment with a rotenone titration cultured in DMEM without pyruvate, with one condition cotreated with 1 mM pyruvate, normalized to start count (n=2). e. Total rotenone ion counts measured by LC-MS 2–5 days post treatment in H1299 cells treated with a rotenone titration in DMEM without pyruvate, with one condition cotreated with 1 mM pyruvate in DMEM (n=2). f. Western blot verifying GOT1/2 double knockout (DKO) in 143B and H1299 cells. GFP antibody also shows that 143B and H1299 cells with jAspSnFR3 (jAsp)/NucRFP express jAspSnFR3 (epitope shared with GFP). Vinculin is used as a loading control. g. Proliferation rates of GOT1/2 DKO 143B cells treated with a titration of environmental aspartate in DMEM without pyruvate (n=3). h. Proliferation rates of GOT1/2 DKO H1299 cells treated with a titration of environmental aspartate for 96 hours in DMEM without pyruvate (n=3). i. GFP/RFP ratio of GOT1/2 DKO H1299 jAspSnFR3/NucRFP cells against a titration of environmental aspartate concentrations for 96 hours in DMEM without pyruvate (n=4). j. NucRFP counts of GOT1/2 DKO H1299 jAspSnFR3/NucRFP cells against a titration of environmental aspartate concentrations in DMEM without pyruvate (n=4). k. GFP/RFP ratio of 143B jAspSnFR3/NucRFP cells treated with Vehicle (DMSO) or 1 μg/mL cycloheximide (CHX) either at the start of the a [file media-1.pdf]

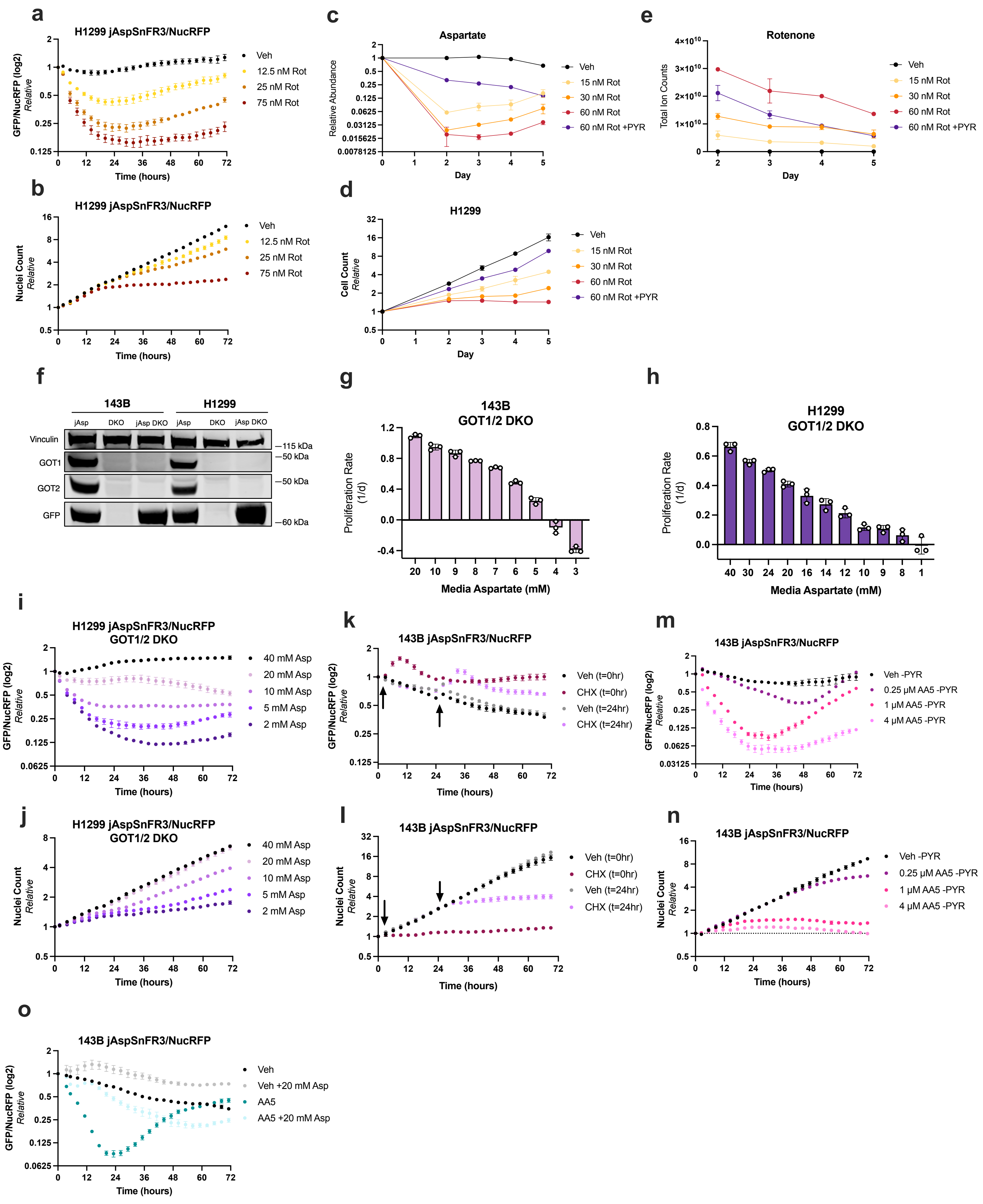

Extended Data Fig. 1

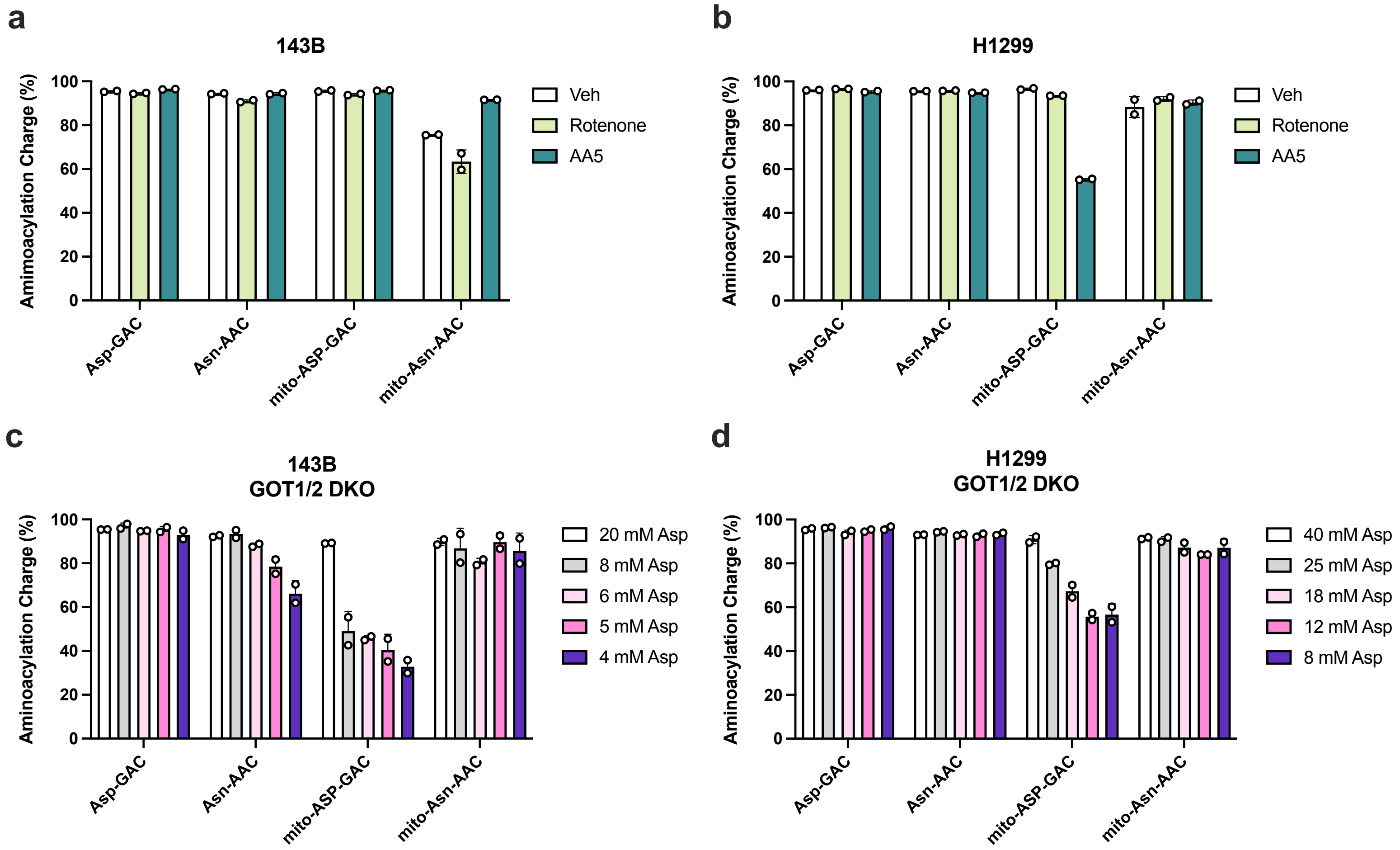

Extended Data Fig. 2

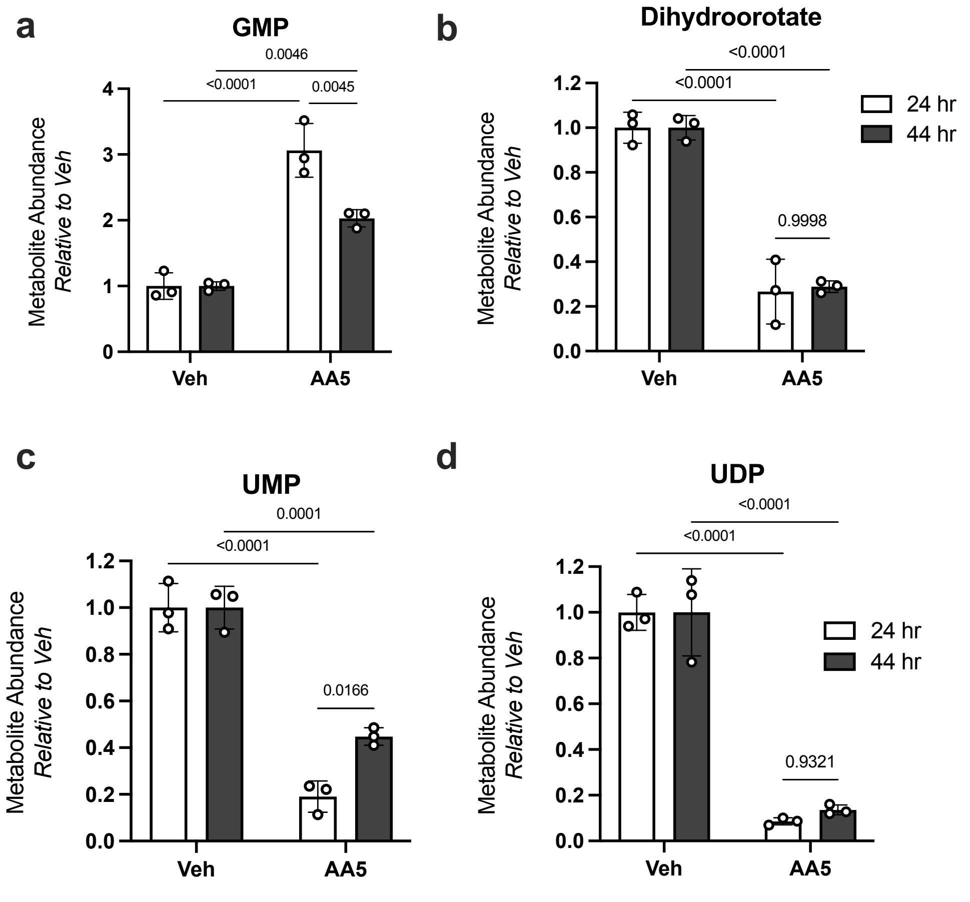

Extended Data Fig. 3

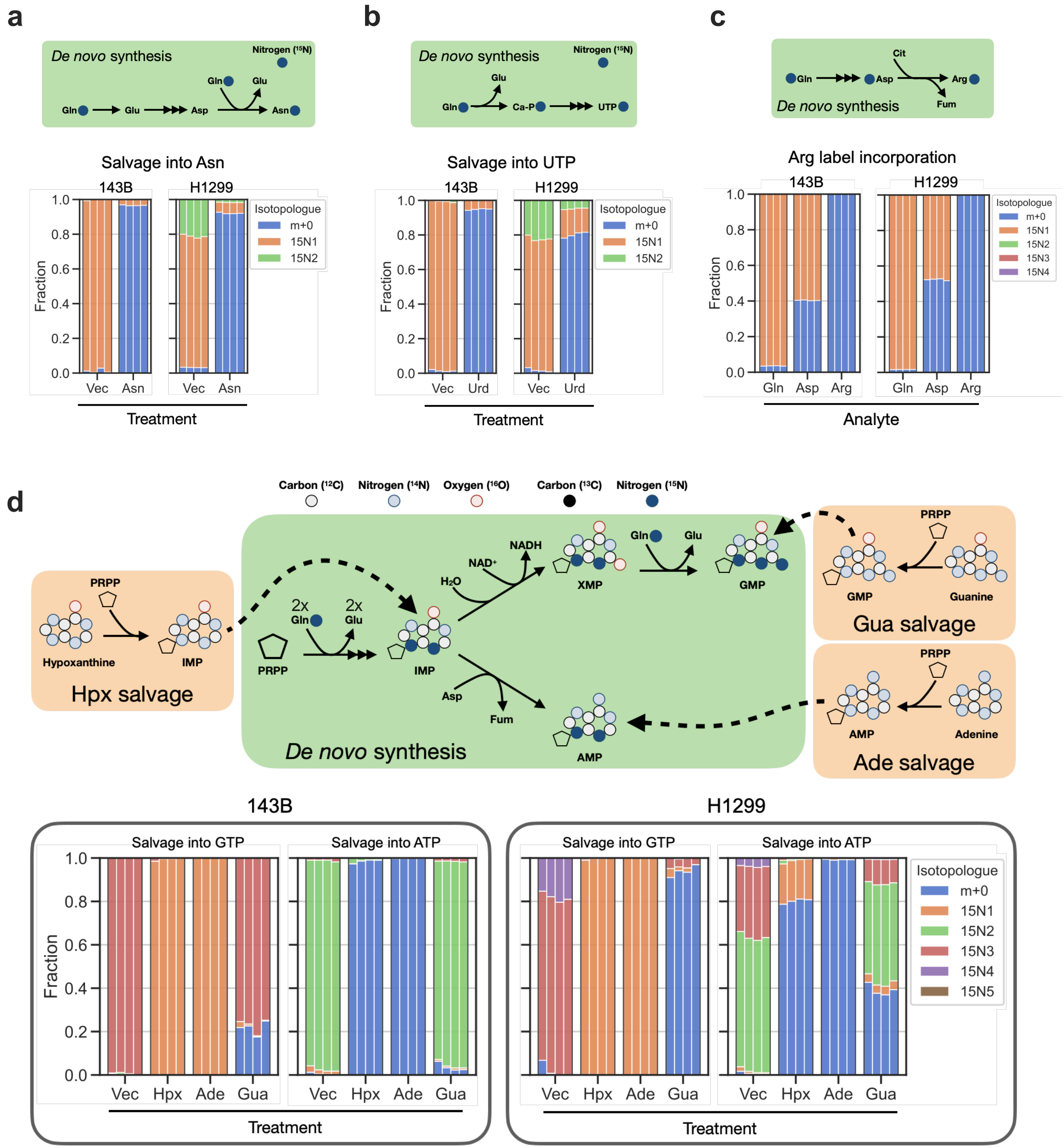

Extended Data Fig. 4

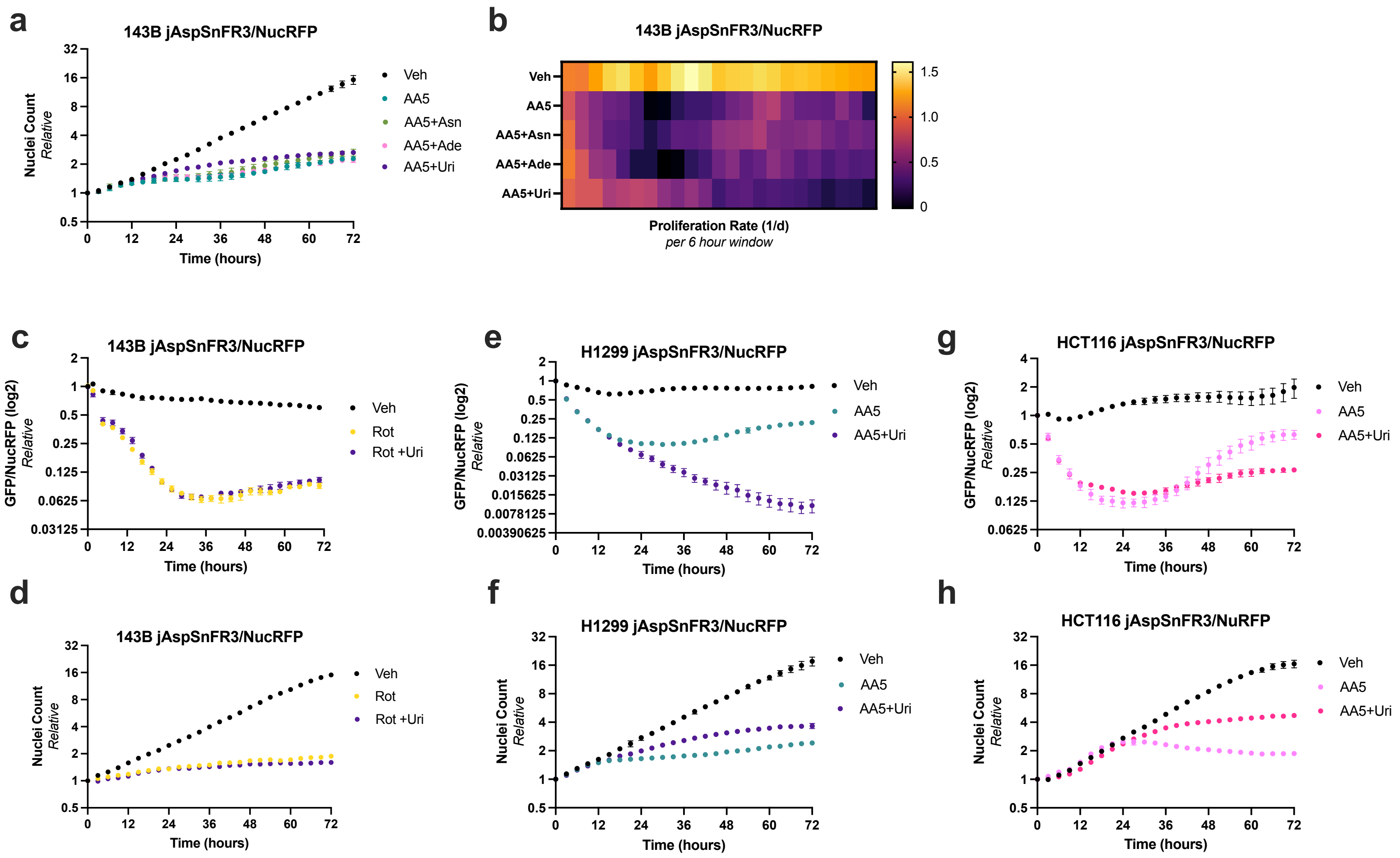

Extended Data Fig. 5

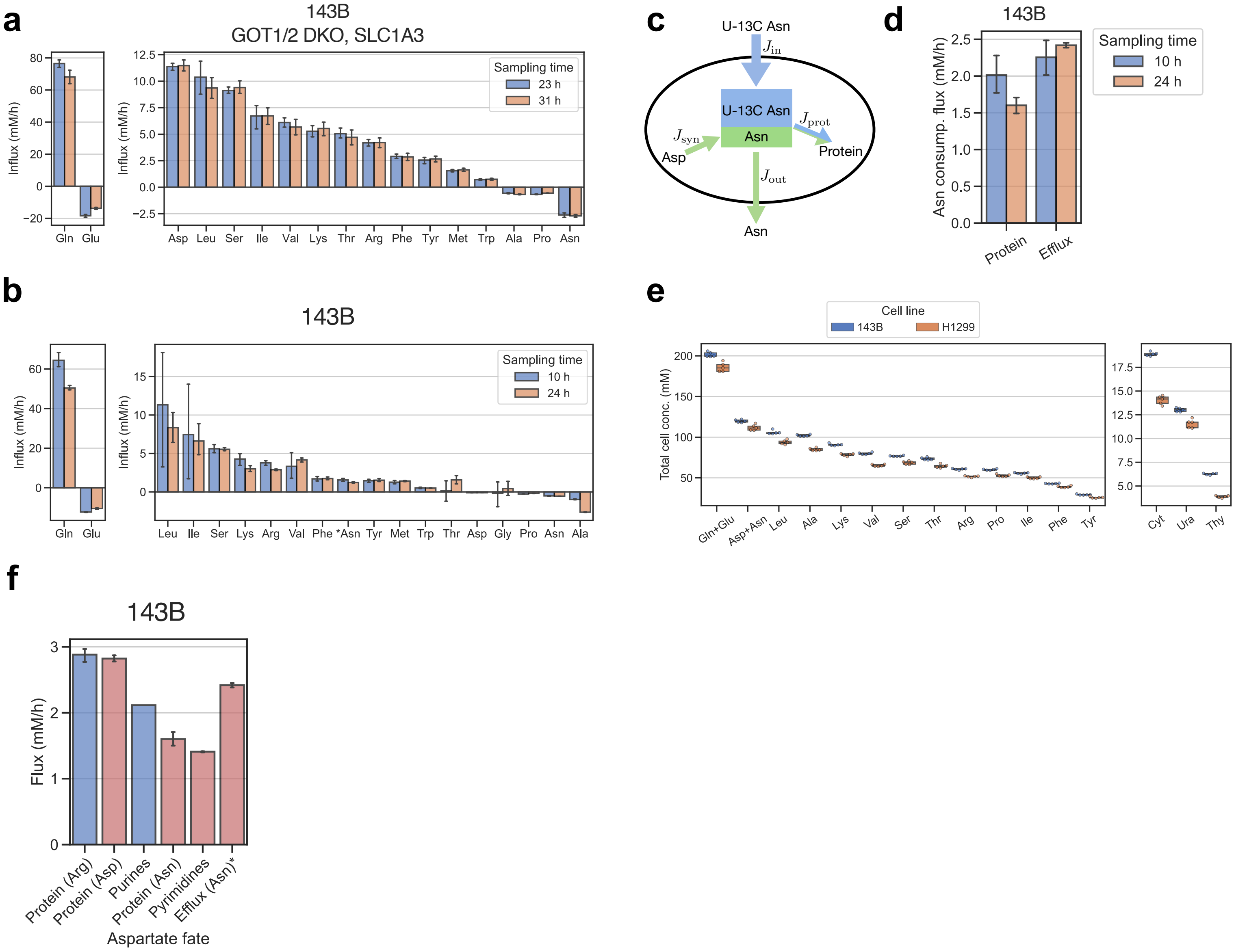

Extended Data Fig. 6

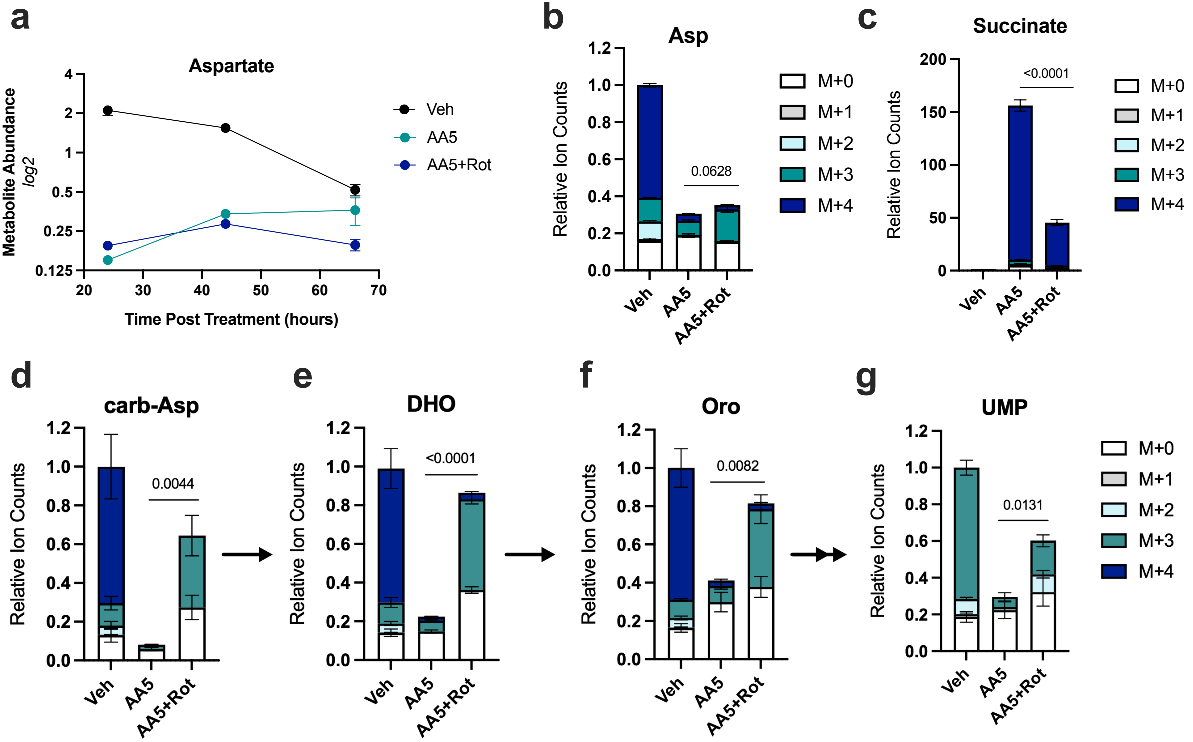

Extended Data Fig. 7

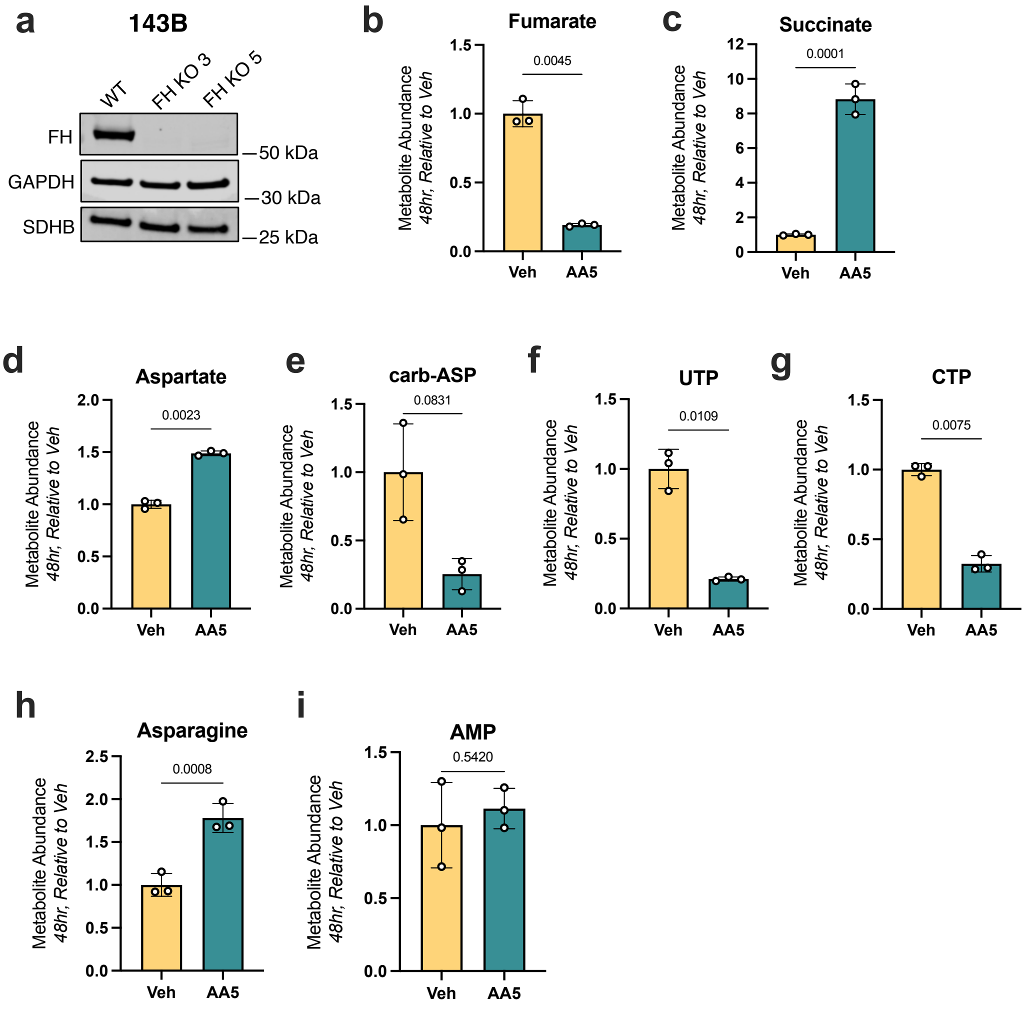

Extended Data Fig. 8

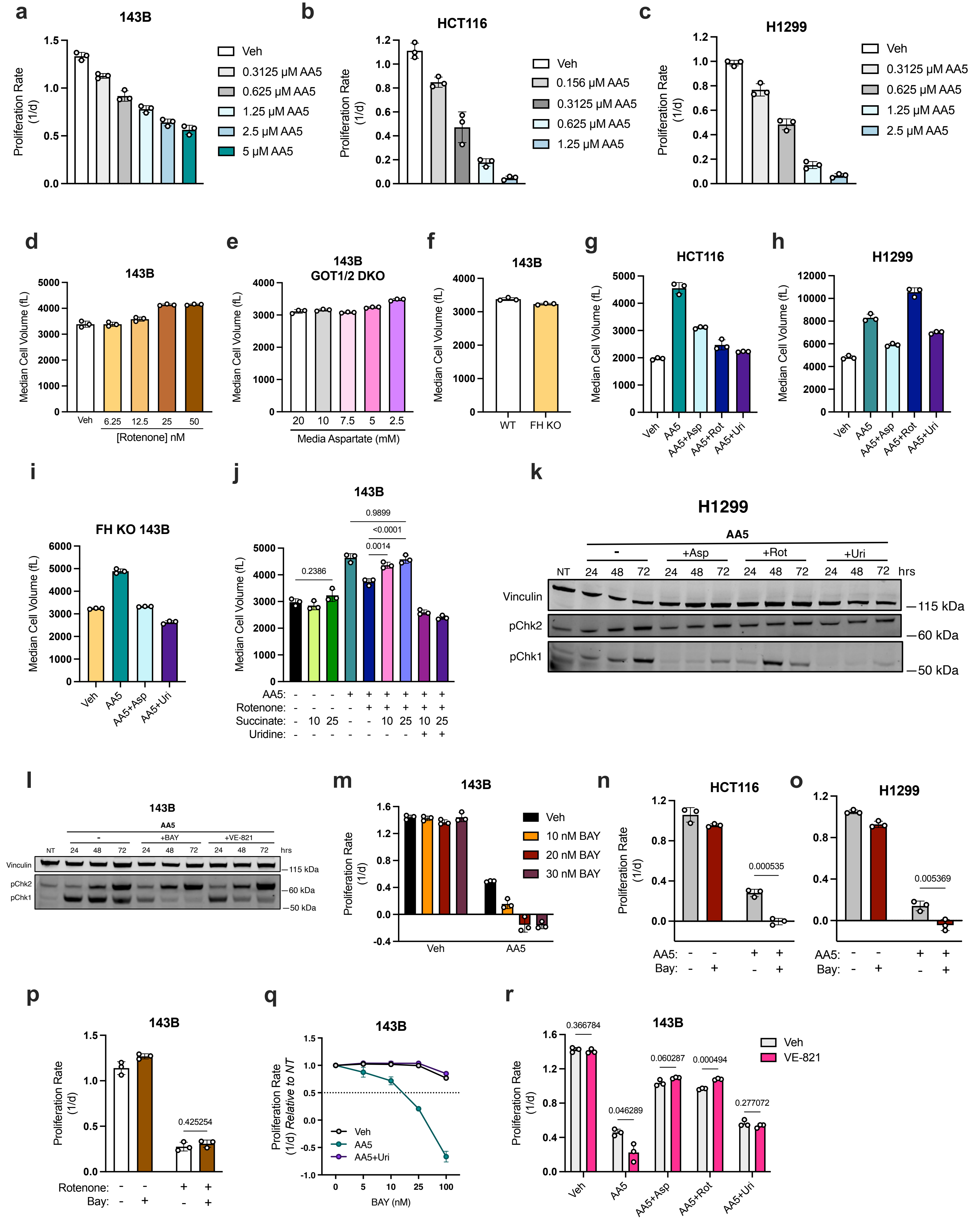

Extended Data Fig. 9
